# Early-Warning Signals of Individual Tree Mortality Based on Annual Radial Growth

**DOI:** 10.3389/fpls.2018.01964

**Published:** 2019-01-08

**Authors:** Maxime Cailleret, Vasilis Dakos, Steven Jansen, Elisabeth M. R. Robert, Tuomas Aakala, Mariano M. Amoroso, Joe A. Antos, Christof Bigler, Harald Bugmann, Marco Caccianaga, Jesus-Julio Camarero, Paolo Cherubini, Marie R. Coyea, Katarina Čufar, Adrian J. Das, Hendrik Davi, Guillermo Gea-Izquierdo, Sten Gillner, Laurel J. Haavik, Henrik Hartmann, Ana-Maria Hereş, Kevin R. Hultine, Pavel Janda, Jeffrey M. Kane, Viachelsav I. Kharuk, Thomas Kitzberger, Tamir Klein, Tom Levanic, Juan-Carlos Linares, Fabio Lombardi, Harri Mäkinen, Ilona Mészáros, Juha M. Metsaranta, Walter Oberhuber, Andreas Papadopoulos, Any Mary Petritan, Brigitte Rohner, Gabriel Sangüesa-Barreda, Jeremy M. Smith, Amanda B. Stan, Dejan B. Stojanovic, Maria-Laura Suarez, Miroslav Svoboda, Volodymyr Trotsiuk, Ricardo Villalba, Alana R. Westwood, Peter H. Wyckoff, Jordi Martínez-Vilalta

**Affiliations:** ^1^Department of Environmental Systems Science, Forest Ecology, Institute of Terrestrial Ecosystems, ETH Zürich, Zurich, Switzerland; ^2^Swiss Federal Institute for Forest, Snow and Landscape Research – WSL, Birmensdorf, Switzerland; ^3^CNRS, IRD, EPHE, ISEM, Université de Montpellier, Montpellier, France; ^4^Institute of Systematic Botany and Ecology, Ulm University, Ulm, Germany; ^5^CREAF, Cerdanyola del Vallès, Catalonia, Spain; ^6^Ecology and Biodiversity, Vrije Universiteit Brussel, Brussels, Belgium; ^7^Laboratory of Wood Biology and Xylarium, Royal Museum for Central Africa, Tervuren, Belgium; ^8^Department of Forest Sciences, University of Helsinki, Helsinki, Finland; ^9^Consejo Nacional de Investigaciones Científicas y Técnicas, CCT Patagonia Norte, Río Negro, Argentina; ^10^Instituto de Investigaciones en Recursos Naturales, Agroecología y Desarrollo Rural, Sede Andina, Universidad Nacional de Río Negro, Río Negro, Argentina; ^11^Department of Biology, University of Victoria, Victoria, BC, Canada; ^12^Dipartimento di Bioscienze, Università degli Studi di Milano, Milan, Italy; ^13^Instituto Pirenaico de Ecología (IPE-CSIC), Zaragoza, Spain; ^14^Centre for Forest Research, Département des Sciences du Bois et de la Forêt, Faculté de Foresterie, de Géographie et de Géomatique, Université Laval, Québec, QC, Canada; ^15^Biotechnical Faculty, University of Ljubljana, Ljubljana, Slovenia; ^16^United States Geological Survey, Western Ecological Research Center, Sequoia and Kings Canyon Field Station, Three Rivers, CA, United States; ^17^Ecologie des Forêts Méditerranéennes (URFM), Institut National de la Recherche Agronomique, Avignon, France; ^18^Centro de Investigación Forestal (CIFOR), Instituto Nacional de Investigación y Tecnología Agraria y Alimentaria, Madrid, Spain; ^19^Institute of Forest Botany and Forest Zoology, TU Dresden, Dresden, Germany; ^20^USDA Forest Service, Forest Health Protection, Saint Paul, MN, United States; ^21^Department of Entomology, University of Arkansas, Fayetteville, AR, United States; ^22^Department of Biogeochemical Processes, Max Planck Institute for Biogeochemistry, Jena, Germany; ^23^Department of Forest Sciences, Transilvania University of Brasov, Brașov, Romania; ^24^BC3 – Basque Centre for Climate Change, Leioa, Spain; ^25^Department of Research, Conservation and Collections, Desert Botanical Garden, Phoenix, AZ, United States; ^26^Faculty of Forestry and Wood Sciences, Czech University of Life Sciences, Prague, Czechia; ^27^Department of Forestry and Wildland Resources, Humboldt State University, Arcata, CA, United States; ^28^Sukachev Institute of Forest, Siberian Division of the Russian Academy of Sciences, Krasnoyarsk, Russia; ^29^Siberian Federal University, Krasnoyarsk, Russia; ^30^Department of Ecology, Universidad Nacional del Comahue, Río Negro, Argentina; ^31^Instituto de Investigaciones en Biodiversidad y Medioambiente, Consejo Nacional de Investigaciones Científicas y Técnicas, Río Negro, Argentina; ^32^Department of Plant and Environmental Sciences, Weizmann Institute of Science, Rehovot, Israel; ^33^Department of Yield and Silviculture, Slovenian Forestry Institute, Ljubljana, Slovenia; ^34^Department of Physical, Chemical and Natural Systems, Pablo de Olavide University, Seville, Spain; ^35^Department of Agricultural Science, Mediterranean University of Reggio Calabria, Reggio Calabria, Italy; ^36^Natural Resources Institute Finland (Luke), Espoo, Finland; ^37^Department of Botany, Faculty of Science and Technology, University of Debrecen, Debrecen, Hungary; ^38^Northern Forestry Centre, Canadian Forest Service, Natural Resources Canada, Edmonton, AB, Canada; ^39^Department of Botany, University of Innsbruck, Innsbruck, Austria; ^40^Department of Forestry and Natural Environment Management, Technological Educational Institute of Stereas Elladas, Karpenisi, Greece; ^41^National Institute for Research and Development in Forestry “Marin Dracea”, Voluntari, Romania; ^42^Departamento de Ciencias Agroforestales, EiFAB, iuFOR – University of Valladolid, Soria, Spain; ^43^Department of Geography, University of Colorado, Boulder, CO, United States; ^44^Department of Geography, Planning and Recreation, Northern Arizona University, Flagstaff, AZ, United States; ^45^Institute of Lowland Forestry and Environment, University of Novi Sad, Novi Sad, Serbia; ^46^Grupo Ecología Forestal, CONICET – INTA, EEA Bariloche, Bariloche, Argentina; ^47^Department of Environmental Systems Science, Institute of Agricultural Sciences, ETH Zürich, Zurich, Switzerland; ^48^Laboratorio de Dendrocronología e Historia Ambiental, Instituto Argentino de Nivología, Glaciología y Ciencias Ambientales, CCT CONICET Mendoza, Mendoza, Argentina; ^49^Boreal Avian Modelling Project, Department of Renewable Resources, University of Alberta, Edmonton, AB, Canada; ^50^Department of Biology, University of Minnesota, Morris, Morris, MN, United States; ^51^Departament de Biologia Animal, de Biologia Vegetal i d’Ecologia, Universitat Autònoma de Barcelona, Cerdanyola del Vallès, Spain

**Keywords:** tree mortality, ring-width, forest, growth, resilience indicators, drought, biotic agents, variance

## Abstract

Tree mortality is a key driver of forest dynamics and its occurrence is projected to increase in the future due to climate change. Despite recent advances in our understanding of the physiological mechanisms leading to death, we still lack robust indicators of mortality risk that could be applied at the individual tree scale. Here, we build on a previous contribution exploring the differences in growth level between trees that died and survived a given mortality event to assess whether changes in temporal autocorrelation, variance, and synchrony in time-series of annual radial growth data can be used as early warning signals of mortality risk. Taking advantage of a unique global ring-width database of 3065 dead trees and 4389 living trees growing together at 198 sites (belonging to 36 gymnosperm and angiosperm species), we analyzed temporal changes in autocorrelation, variance, and synchrony before tree death (diachronic analysis), and also compared these metrics between trees that died and trees that survived a given mortality event (synchronic analysis). Changes in autocorrelation were a poor indicator of mortality risk. However, we found a gradual increase in inter-annual growth variability and a decrease in growth synchrony in the last ∼20 years before mortality of gymnosperms, irrespective of the cause of mortality. These changes could be associated with drought-induced alterations in carbon economy and allocation patterns. In angiosperms, we did not find any consistent changes in any metric. Such lack of any signal might be explained by the relatively high capacity of angiosperms to recover after a stress-induced growth decline. Our analysis provides a robust method for estimating early-warning signals of tree mortality based on annual growth data. In addition to the frequently reported decrease in growth rates, an increase in inter-annual growth variability and a decrease in growth synchrony may be powerful predictors of gymnosperm mortality risk, but not necessarily so for angiosperms.

## Introduction

Episodes of tree mortality associated with drought and heat stress have been reported in many forested biomes over the last decades (Allen et al., 2010; Hartmann et al., 2018), and are expected to increase under ongoing climate change in many regions (Allen et al., 2015). Forest dieback can induce multiple changes in forest functions and dynamics (Franklin et al., 1987; Anderegg et al., 2013a, 2016b), including rapid shifts in vegetation composition (Martínez-Vilalta and Lloret, 2016) or significant changes in terrestrial carbon sequestration with resulting feedbacks to the climate system (e.g., Carvalhais et al., 2014). In addition to the direct loss of individuals, tree mortality may also reduce forest regeneration capacity by decreasing the number of potential reproductive individuals, and by modifying the micro-environmental conditions and biotic interactions (e.g., Mueller et al., 2005; Royer et al., 2011). Being able to forecast when and where tree mortality episodes are likely to occur is thus a prerequisite for effective and adaptive forest management, especially under progressively warmer and drier conditions (Pace et al., 2015; Trumbore et al., 2015).

Evaluating individual tree mortality risk requires reliable indicators that reveal temporal changes in tree vitality (Allen et al., 2015; Hartmann et al., 2018). Such information can be provided by physiological and anatomical data. Both abrupt and long-term declines in hydraulic conductivity caused by drought-induced xylem embolism (Anderegg et al., 2013b; Adams et al., 2017; Choat et al., 2018) or changes in wood anatomical features (e.g., lower lumen area; Hereş et al., 2014; Pellizzari et al., 2016) may indicate impending tree death. In association with low whole-plant conductivity, reduced carbon assimilation and depletion of stored carbohydrates may also occur due to the decline in stomatal conductance and leaf area, particularly for gymnosperms (Galiano et al., 2011; Pangle et al., 2015; Adams et al., 2017). The determination of such mechanistic indicators is, however, costly, and temporally and spatially limited. Therefore, other approaches have been used to identify changes in tree health and mortality risk, such as temporal changes in crown defoliation (Dobbertin and Brang, 2001), or in radial growth rates (e.g., Pedersen, 1998; Bigler and Bugmann, 2004; Dobbertin, 2005; Camarero et al., 2015; Hülsmann et al., 2018). Ring-width (RW) data are especially suitable, as they provide retrospective and long-term information about tree radial growth at an annual resolution, and can be applied effectively at an affordable cost to a large number of trees, sites, and species.

A recent synthesis reported either abrupt or long-term reduction in growth rates before death in most tree mortality events recorded in dendrochronological studies worldwide (Cailleret et al., 2017). However, this decrease in growth before mortality was not ubiquitous, and its detection was subject to important methodological constraints, especially related to the sampling design (Cailleret et al., 2016). Therefore, additional metrics that go beyond changes in absolute growth rates are needed to identify individuals at high risk of mortality. Early-warning signals (EWS) have been proposed to characterize (ecological) systems that are approaching a critical transition, i.e., a sudden and persistent shift in a system’s state (Scheffer et al., 2009). EWS are caused by the gradual decrease in the recovery rate of a system after a perturbation – called “critical slowing down” (Wissel, 1984) – and have been identified prior to population extinction in experiments under increasing levels of stress (e.g., Drake and Griffen, 2010; Dai et al., 2012; Veraart et al., 2012). Tree death can be considered as system failure (Anderegg et al., 2012), and can be viewed as a critical transition caused by the combined changes in the intensity, frequency and duration of stress factors (Dakos et al., 2015), and high sensitivity of the tree to these specific stresses (Brandt et al., 2017). This would be somewhat analogous to recent applications of critical transitions theory to human physiology, where health failures at the individual level can be anticipated with EWS (Olde Rikkert et al., 2016). In fact, the growth rate decline observed in most trees before mortality may be typical of such “critical slowing down” phenomenon, which can be captured by an increase in temporal autocorrelation and variance in time series of variables reflecting the functioning of the system (Scheffer et al., 2009; Dakos et al., 2012b), and by a decrease in their synchrony with the environment. These EWS would, respectively, reveal that the state of the system at any given moment becomes more and more like its recent past state, increasingly affected by shocks, and less able to track the environmental fluctuations (Scheffer et al., 2009).

Several studies have reported that RW time series of dying or declining individual trees tend to show increasing temporal autocorrelation and variance over time or higher values than surviving individuals (e.g., Ogle et al., 2000; Suarez et al., 2004; Millar et al., 2007; Kane and Kolb, 2014; Camarero et al., 2015; see Supplementary Appendix A), especially in the case of drought-induced mortality (McDowell et al., 2010; Heres et al., 2012; Gea-Izquierdo et al., 2014; Macalady and Bugmann, 2014). However, it remains unclear whether rising growth variance and autocorrelation can be used as EWS for tree mortality. First, other studies have reported opposite trends (e.g., Pedersen, 1998; Millar et al., 2012), or contrasting results depending on the study species (Camarero et al., 2015), sites (Ogle et al., 2000), and tree size (Herguido et al., 2016). Second, finding a common trend comparing results across different case studies can be difficult, as methodologies vary among studies, especially for the quantification of the inter-annual variability in growth. This aspect is fundamental, as opposite relationships could be obtained when using the standard deviation (SD) or the mean sensitivity (i.e., the mean relative change in RW between two consecutive rings; see Bunn et al., 2013) to characterize year-to-year variability in RW series (Gillner et al., 2013; Macalady and Bugmann, 2014). Similarly, Camarero et al. (2015) did not find any consistent change in growth synchrony between declining and healthy trees among species.

Here, we tested whether EWS based on annual radial growth data can be used as universal indicators of tree mortality. We used a unique, pan-continental database that contains paired growth time series for dead and surviving trees from nearly 200 sites, including data for 13 angiosperm and 23 gymnosperm species. In particular, we measured temporal changes in tree growth variance, temporal autocorrelation, and synchrony (correlation among trees) after removing any effect driven by changes in absolute growth rates, which had been studied in a previous publication (Cailleret et al., 2017). We analyzed temporal changes in the properties of RW chronologies of individual trees that died during a given stress event (diachronic approach on dying trees), and compared the resulting patterns to those from trees that survived this specific event (synchronic approach). Contrary to standard tree growth analysis that explores trends in RW chronologies, our approach here is to estimate changes in the dynamic properties of these time series (e.g., autocorrelation structure) that can be used as proxies of tree mortality risk. The methodology we develop may assist in using such proxies for assessing individual tree resilience.

## Materials and Methods

### Tree-Ring Width Chronologies

We used the pan-continental tree-ring width (mm) database compiled by Cailleret et al. (2017), which includes 58 published and unpublished datasets for which (i) both dying and surviving trees growing together at the same site were cored, (ii) all individual chronologies had been successfully cross-dated, (iii) mortality was proximally induced by stress (e.g., drought, competition, and frost) and biotic agents in an endemic phase (e.g., bark beetles, defoliator insects, fungi, acting as predisposing or contributing factor), and not by abrupt abiotic disturbances such as windthrow, fire, or flooding, which may kill trees irrespective of their vitality and previous growth patterns (but see Nesmith et al., 2015). We grouped the datasets into four groups according to the main mortality sources determined by the authors of each study: (i) ‘drought’ corresponds to mortality caused by a single or several drought events without obvious impact of biotic agents; (ii) ‘biotic’ includes sites in which mortality was induced primarily by biotic factors, including bark-beetles, defoliator insects, and/or fungal infection; (iii) ‘drought and biotic’ when the impact of biotic agents (including mistletoes and wood-borers) was associated with drought; (iv) and the group ‘others’ includes snow break, frost events, high competition intensity, and cases in which mortality were not evident or not specified.

The database analyzed here slightly differs from Cailleret et al. (2017) as some sites for which we previously did not find any pair of dying/surviving tree with similar diameter at breast height (DBH) are considered in the present analysis, and as we excluded trees with less than 20 measured rings (see below). A total of 36 gymnosperm and angiosperm species were studied, with an overrepresentation of gymnosperms (64% of the species and 86% of the sites). Pinaceae was the most represented family, followed by Fagaceae. Overall, the dataset analyzed in the main text included 3065 dead trees and 4389 living trees growing at 198 sites mostly in boreal, temperate, and Mediterranean biomes of North America and Europe. More details on the sampling methods and on the assessments of the mortality sources, tree cambial age, DBH, and the year of death are available in Supplementary Appendix B and in Cailleret et al. (2017).

### Growth Metrics

**FIGURE 1:**
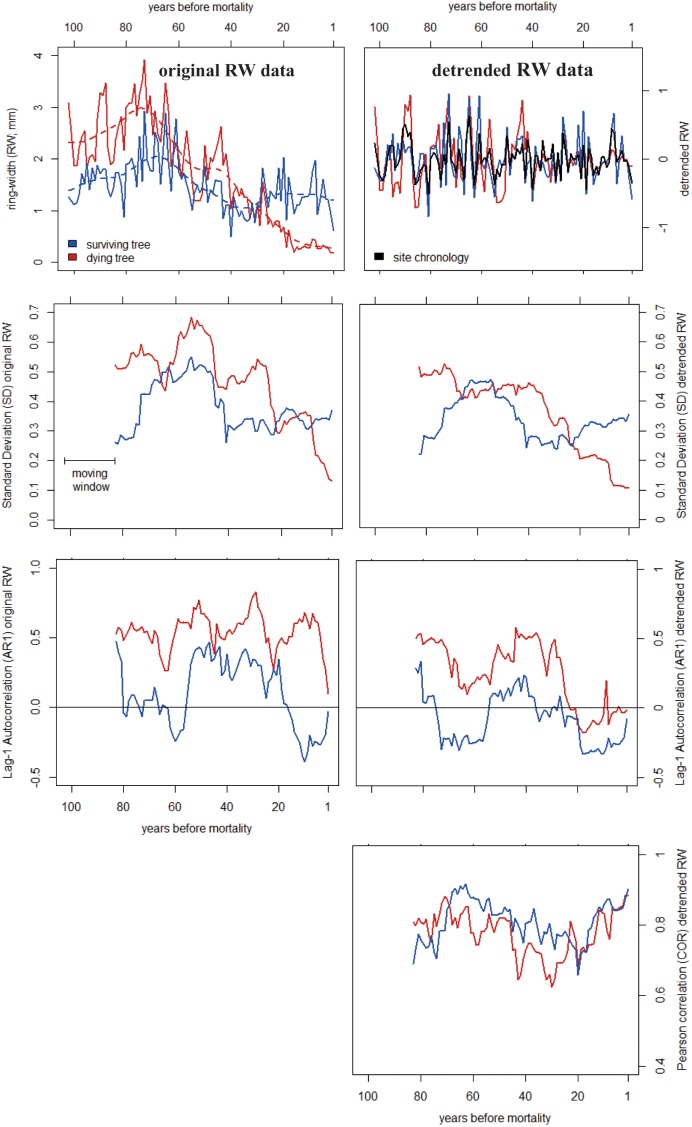
Example of early-warning signals of tree mortality based on ring-width (RW) data from two *Abies alba* trees from Mont Ventoux, France (Cailleret et al., 2014). The Standard Deviation (SD), first-order autocorrelation (AR1) and Pearson correlation coefficients (COR) were calculated on the original (*Left*) and detrended (*Right*) RW data using 20-year moving time windows.

**FIGURE 2:**
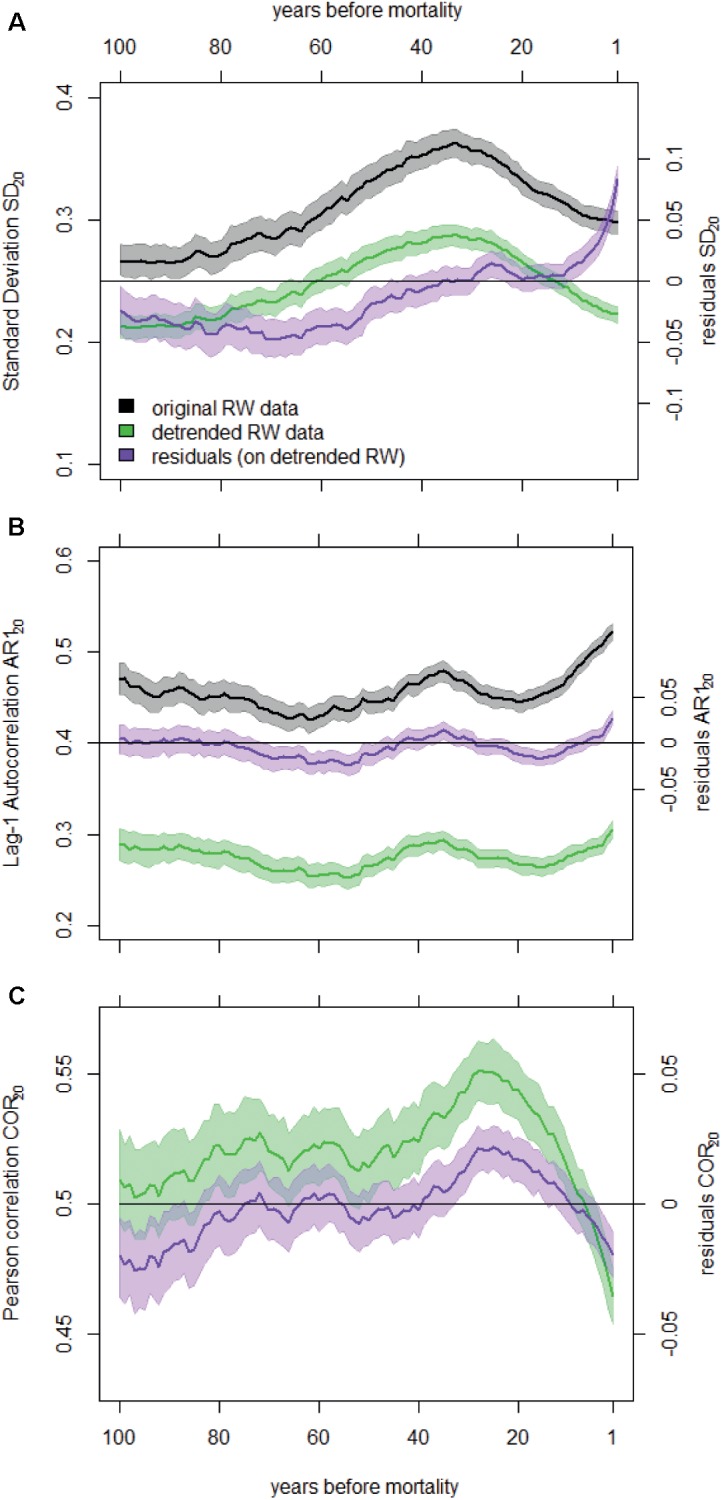
Temporal change in SD_20_ **(A)**, AR1_20_ **(B)**, and COR_20_ **(C)** before death averaged for all dying trees and calculated on the original and detrended RW data. We also show the temporal change in the residuals of the linear mixed-effects models fitted to these metrics (right *y*-axes). Shaded areas represent the 95% confidence intervals of the means. Note that COR20 values were not calculated on not-detrended RW data.

Following Dakos et al. (2012a) and Camarero et al. (2015), we estimated levels and trends of Standard Deviation (SD) and first-order autocorrelation (AR1) in detrended RW time series of individual trees (Figure 1). Contrary to most dendrochronological studies, where AR1 is calculated using raw RW time series (e.g., Martín-Benito et al., 2008; Esper et al., 2015; Hartl-Meier et al., 2015), chronologies were detrended to correct for decadal to centennial trends, including decadal decreases in growth rates that are commonly observed prior to mortality (Cailleret et al., 2017). Such negative growth trends would automatically lead to increasing trends in AR1 before tree death (Figure 2B and Supplementary Appendix C), irrespective of the potential intrinsic change in the AR1 properties related to changes in tree vitality. In addition, we calculated the Pearson correlation (COR) coefficient between individual time series and the site chronology (Figure 1). In contrast to the study by Camarero et al. (2015), where COR coefficients corresponded to the correlations between separated mean chronologies of ‘declining’ and ‘non-declining’ trees, we analyzed COR values between each individual detrended time series of dying trees and the corresponding site- and species-specific chronology (including both dying and surviving trees), to reduce potential biases at sites where few living trees had been sampled. Site chronologies were derived using the bi-weight robust mean of the individual residual chronologies (Figure 1) to reduce the importance of outliers. This is particularly important when sample size is low, which is the case for some of our sites (Supplementary Appendix B).

As we aimed at analyzing temporal changes in growth SD and AR1, and at comparing them among trees with different ages, sizes, or growth rates, two precautionary measures were taken to detrend the RW data. (1) Most tree-ring- based studies remove size-effects on the RW data while keeping climate-induced decadal to centennial changes in growth rates using negative exponential curves or using the Regional Curve Standardization method (e.g., Peters et al., 2015; Büntgen et al., 2017). In contrast, we used smoothing splines which are more flexible and more adapted to remove decadal trends (Cook and Peters, 1997). As SD and AR1 values are highly sensitive to the bandwidth of the Gaussian kernel regression (see Supplementary Appendix D), this one was fixed at 15 years rather than proportional to the length of the time-series. Indeed, the latter approach would bias the comparison among trees with different length of the time-series (∼different ages). As we specifically focused on the end of the RW time series, our analysis is prone to edge-effects that can emerge from Gaussian detrending (e.g., D’Arrigo et al., 2008; see Supplementary Appendix E). Thus, the sensitivity of our results to the bandwidth length was also assessed (Supplementary Appendix D). (2) We used residuals (differences between the original (raw) RW data and the smoothing spline from the Gaussian kernel regression) rather than ratios as done in traditional dendrochronological studies. In this way, the output chronology is centered on zero, is still heteroscedastic, and does not include annual outliers when RW is close to zero, which often occurs in dying trees. In contrast, most dendrochronological studies using RW data calculate ratios to get series that are centered on one and are assumed to be homoscedastic (see Cook and Peters, 1997; Büntgen et al., 2005; Frank et al., 2006; Supplementary Figure C2). To detect short-term (∼decadal) but still robust changes in growth metrics, SD, AR1 and COR were calculated within a 20-year moving time-window (hereafter SD_20_, AR1_20_, and COR_20_). Trees with fewer than 20 rings were thus discarded from this analysis. Other lengths of the moving time-window were tested and showed similar results (Supplementary Appendix F).

### Detecting Trends in Growth Metrics Before Tree Mortality

Our dataset allowed us to follow two approaches for estimating EWS that helped us to increase the robustness of our conclusions and to assess potential methodological biases. The first approach was based on the analysis of the temporal changes in growth patterns of dying trees (diachronic approach), and the second on the comparison between dying and surviving individuals coexisting at the same site (synchronic approach).

#### Temporal Change in Growth Metrics of Dying Trees

For each of the 3065 dying trees, we calculated SD_20_, AR1_20_, and COR_20_ until the last year with complete ring formation, i.e., the year before tree death. We determined whether absolute values in SD_20_, AR1_20_, and COR_20_ calculated during the last 20 years preceding mortality (SD_20*f*_, AR1_20*f*_, and COR_20*f*_ for final values) were significantly different than those during any other previous 20-year period.

As SD_20_ calculated on the detrended chronology was still positively related to mean growth rate calculated over the same period (meanRW_20_; see Supplementary Appendix C), we did not directly analyze this metric, but instead we analyzed the residuals of a linear mixed-effect model (LMM) fitted to the overall dataset with meanRW_20_ as a fixed explanatory variable. The same approach was used for AR1_20_ and COR_20_ to center them on zero, which allows for an easier comparison among trees, species, and periods with different mean growth rates. This is especially important as our sampling is not equal in terms of mean tree age per species, which could lead to problems when averaging results to analyze the overall temporal dynamics in growth metrics. Bootstrap resampling procedures were then used to test if the LMM residuals for SD_20*f*_, AR1_20*f*_, and COR_20*f*_ significantly differed from zero (500 re-samplings).

SD_20_ and meanRW_20_ were log-transformed unlike AR1_20_ and COR_20_ values because their distributions were normal. As each tree species may have different SD and AR1 values for a similar growth rate (e.g., higher AR1 values are expected for evergreen species; Anderegg et al., 2015b), and COR values may depend on the number of trees used to derive the reference chronology, random effects were estimated for the intercept and the slope with species crossed with site as a grouping factor.

#### Differences in Growth Metrics Between Conspecific Dying and Surviving Trees

Although RW data were detrended using Gaussian filtering before calculating SD_20_, AR1_20_, and COR_20_, temporal changes in these metrics could be affected by site-specific decadal-scale changes in environmental conditions (e.g., change in climatic conditions or in canopy dynamics; Brienen et al., 2006; Carrer and Urbinati, 2006; Esper et al., 2015), regardless of individual intrinsic changes in tree vitality. Thus, to account for this possibility, we compared SD_20*f*_, AR1_20*f*_, and COR_20*f*_ between conspecific dying and surviving trees for each mortality event, i.e., for each combination of species, site, and mortality year (see Cailleret et al., 2017).

For each dying tree, two approaches were followed for selecting comparable conspecific surviving trees from the same site: we only considered trees (i) with a similar DBH at the given mortality year (difference in final DBH between dying and surviving trees diff_D-S_DBH_f_ ≤ 2.5 cm), or (ii) with a similar mean RW during the 20-year period before the mortality year (diff_D-S_meanRW_20*f*_ ≤ 5%). In cases where none of the surviving trees fulfilled this condition, the corresponding dying tree was discarded. Following these two approaches, we considered 2887 (94.2% of the dying trees) and 2093 (68.3%) pairs of trees, respectively. On the one hand, comparing trees with similar DBH removes both geometric and structural (∼size) effects (see Bowman et al., 2013). For instance, large and dominant trees tend to show more plastic growth than small and suppressed ones (Martín-Benito et al., 2008; Mérian and Lebourgeois, 2011). On the other hand, comparing trees with similar mean RW removes mathematical effects related to changes in growth rate (see Supplementary Appendix C), and allows us to detect the presence of growth-based EWS in case of unchanging growth level before tree death (relative to the surviving trees). Thus, these two sampling approaches may individually bias the results, but they are complementary and should be considered together.

On both datasets, we analyzed if the differences in SD_20*f*_, AR1_20*f*_, and COR_20*f*_ between conspecific dying and surviving trees (diff_D-S_SD_20*f*_, diff_D-S_AR1_20*f*_, and diff_D-S_COR_20*f*_) were significantly different from zero for all species groups and mortality sources using LMMs and bootstrapping methods. For each of these response variables, we fitted a LMM considering the species group and mortality source as interactive fixed effects. As size or geometric effects could remain, we also included the difference in final mean RW (diff_D-S_RW_20*f*_) and in DBH (diff_D-S_DBH_f_) as fixed effects. Random effects were estimated for the intercept with species crossed with site as grouping factor. Direct age effects were not considered here assuming that senescence only marginally affects tree function (Mencuccini and Munné-Bosch, 2017). LMMs were finally used to predict diff_D-S_SD_20*f*_, diff_D-S_AR1_20*f*_, and diff_D-S_COR_20*f*_ values in the theoretical situation in which dying trees have similar meanRW_20*f*_ and DBH_f_ as surviving ones.

#### Sampling Scheme

To account for the heterogeneity in the number of dying trees per site and per species in the dataset, we used two resampling procedures (Cailleret et al., 2017). First, we randomly sampled with replacement the same number of dying trees (diachronic approach) or the same number of dying-surviving pairs (synchronic approach) for each of the 36 species. Second, a similar approach was followed to provide the same weight in the calibration dataset for each of the 198 sites. With both approaches, each species or each site contributes equally to the results, which minimizes the bias related to under-sampling or over-sampling of specific sites or species (Supplementary Appendix G).

#### Theoretical Expectations

Finally, to detect which combinations of temporal trends in SD and AR1 can be expected when growth rates gradually decrease (commonly reported for dying trees), we generated theoretical RW time series based on simple growth models that included (i) an autocorrelation component, (ii) a long-term change in the mean, and (iii) some noise reflecting the environmental stochasticity (Supplementary Appendix E).

The calculation of moving SD_20_, AR1_20_, and COR_20_ values, and LMM analyses were performed using the packages *earlywarnings* (Dakos et al., 2012a), *lme4* (Bates et al., 2014), and *lmerTest* (Kuznetsova et al., 2017) of the open-source software R (R Core Team, 2017).

## Results

### Temporal Changes in Growth Metrics of Dying Trees

**FIGURE 3:**
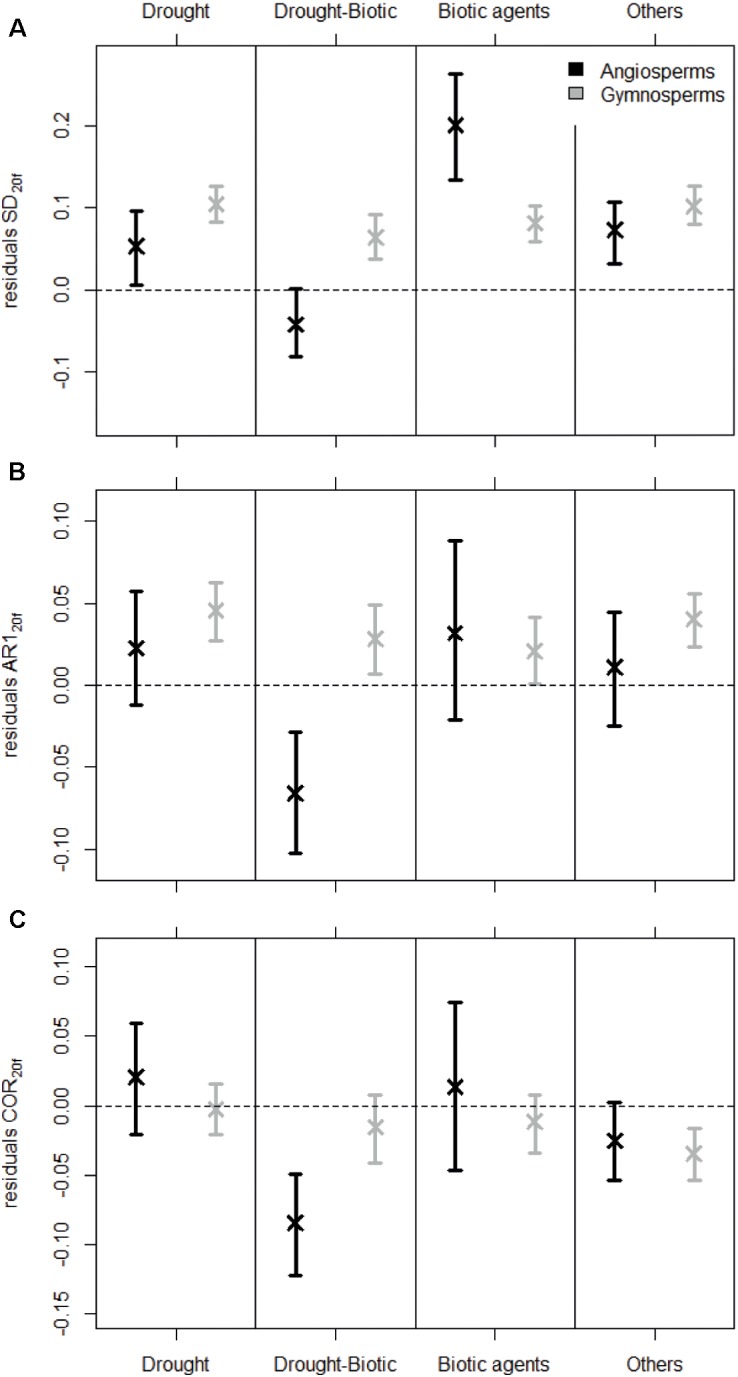
Variation in the residuals of SD **(A)**, AR1 **(B)**, and COR **(C)** calculated over the last 20-year period of the detrended ring-width time series preceding tree death (resSD_20*f*_, resAR1_20*f*_, and resCOR_20*f*_) among mortality sources and species groups. Error bars depict 95% confidence intervals of the mean residuals, which were determined from 500 bootstrap resamplings of the original dataset.

SD_20_ calculated on detrended RW data started decreasing around 30 years before tree death (Figure 2A). This trend in SD_20_ was related to the general reduction in mean RW, as both variables are highly correlated (Supplementary Appendix C). After removing the effect of the mean RW using a LMM, SD residuals revealed an increase in inter-annual variability of RW before trees died (Figure 2A). The variability calculated for the 20-year period before mortality (resSD_20*f*_) was generally higher than during the rest of the lives of dying trees (Figure 3). For gymnosperms, this pattern was significant irrespective of the mortality cause and of the method used to account for the heterogeneity in sample properties (Figure 3A and Supplementary Appendix G). In addition, the increase in variability was even stronger in the last 10-year period before mortality (Supplementary Appendix F). Results were less clear for angiosperms. Although variability was generally significantly higher at the end of an angiosperm’s life, this pattern was not present for all sources of mortality (e.g., when mortality was caused by both drought and biotic agents, Figure 3A), and resSD_20_ did not monotonically increase toward the end of a tree’s life (Supplementary Figure G1B).

The first-order autocorrelation increased on average before tree death both in detrended RW chronologies (AR1_20_) and in the residuals of the LMMs (resAR1_20_) (Figure 2B). In fact, the residual AR1 (after removing both growth level and trend effects, Supplementary Appendix C) was higher than zero in the final 20-year period preceding tree death (resAR1_20*f*_; Figure 3B). However, this was mostly true for gymnosperms (except when mortality was caused by both drought and biotic agents in samples including equal number of dying trees per species; Supplementary Appendix G), and such level of positive resAR1_20_ values was not exclusive to the end of a gymnosperm’s life (Supplementary Figure G1C). Thus, the high AR1 values calculated during the 20-year period before gymnosperm mortality should not be interpreted as an exclusive response indicative of impending tree death. In the case of angiosperms, no significant or monotonic change in resAR1_20_ was observed consistently before mortality (Figure 3B and Supplementary Figure G1D).

On average, Pearson correlations calculated between individual RW time series of dying trees and site chronologies decreased gradually ca. 30 years before death (Figure 2C). However, residual correlation values (resCOR_20_; after correcting for mean RW, Supplementary Appendix C) were not consistently below zero or lower than any previous period across mortality sources, species groups, or sampling strategies (Figure 3C and Supplementary Appendix G). The only exceptions were mortality caused by both drought and biotic agents for angiosperms and mortality caused by other factors in gymnosperms (Figure 3C and Supplementary Figure G2).

### Differences in Temporal Changes of Growth Metrics Between Conspecific Dying and Surviving Trees

**FIGURE 4:**
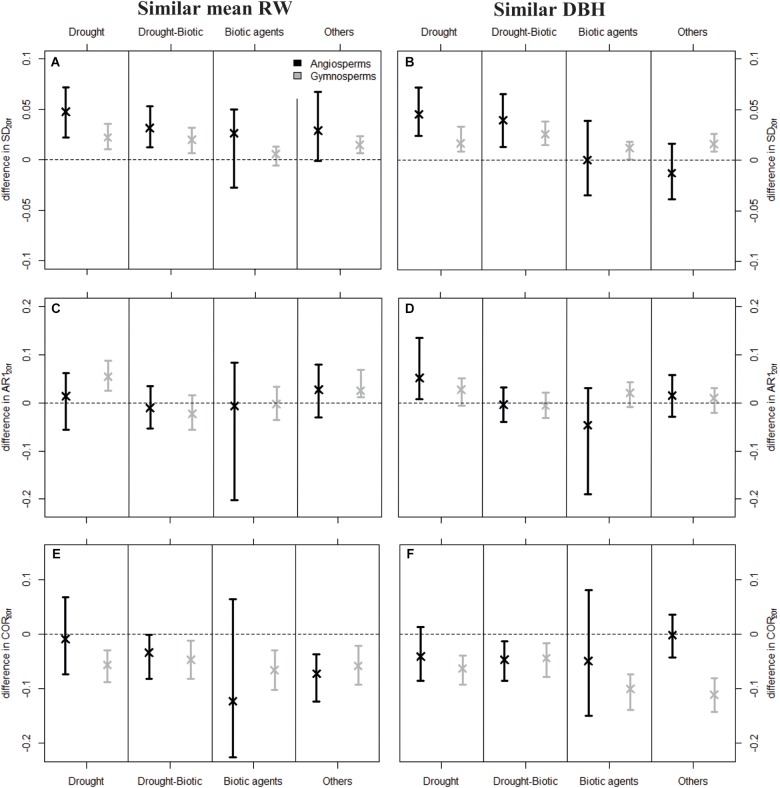
Mean difference in SD_20*f*_ **(A,B)**, AR1_20*f*_ **(C,D)**, and COR_20*f*_ **(E,F)** values between dying and surviving trees predicted by the linear mixed-effects models (LMMs) fitted to the original dataset, fixing diff_D-S_RW_20*f*_ and diff_D-S_DBH_f_ at zero. Positive values mean that dying trees showed higher SD_20*f*_, AR1_20*f*_, or COR_20*f*_ compared to conspecific surviving trees. Standardization was based on similar meanRW_20*f*_ (*Left*) and similar DBH_f_ (*Right*). Error bars depict 95% confidence intervals of the predicted mean differences, which were determined from 500 bootstrap resamplings. Estimates of the LMMs are available in Supplementary Table H1.

Dying trees generally showed higher variability in growth in the last 20 years of their lives compared to surviving trees. Estimated differences in variance between dying and surviving trees (diff_D-S_ SD) based on LMMs adjusted for growth rate (meanRW_20*f*_) and size effects (DBH_f_) were significantly higher than zero in most cases for both angiosperms and gymnosperms and across mortality drivers, except when trees were killed by biotic agents (Figures 4A,B). This result was generally robust to different sampling schemes (unbalanced original dataset in Figure 4 vs. equal weight among species or sites in Supplementary Appendix G). Dying gymnosperms showed more consistent effects, although the magnitude of the SD difference between dying and surviving trees was generally higher for angiosperms (Figures 4A,B).

Contrary to variance, autocorrelation did not significantly differ between dying and surviving trees. In specific cases, differences were significantly higher than zero (e.g., gymnosperms for drought-induced mortality and pairing by meanRW_20*f*_), but this was never consistent across mortality drivers or sampling schemes (Figures 4C,D and Supplementary Appendix G).

Finally, we found predominantly lower COR_20*f*_ for dying trees than surviving ones (Figures 4E,F). This pattern was largely consistent and of similar magnitude for every mortality source for gymnosperms, but it was less clear for angiosperms, as some differences in correlation (e.g., when biotic agents were the main mortality source) strongly depended on the sampling strategy, i.e., on the species and sites considered (Supplementary Appendix G).

## Discussion

We found a gradual increase in inter-annual growth variability and a decrease in growth synchrony during the ∼20-year period before mortality. These trends were more robust for gymnosperms than for angiosperms, irrespective of the main cause of mortality. However, this result only partly conforms to the patterns that are expected to characterize systems prior to transitions due to critical slowing down (Scheffer et al., 2009; Dakos et al., 2012b), as no consistent changes in growth autocorrelation was detected for either taxonomic group.

### Mechanisms Underlying the Differences Between Angiosperms and Gymnosperms

The increase in growth variance (for a given growth level) of dying gymnosperms may indicate an increase in susceptibility to external influences such as climatic factors or pathogen diseases (e.g., Csank et al., 2016; Timofeeva et al., 2017). In addition, their growth seems to be less coupled to high-frequency climate fluctuations than surviving gymnosperms, as revealed by the decrease in growth synchrony with the overall site chronology (Fritts, 1976; Boden et al., 2014). Both changes may be associated with small-scale differences in atmospheric conditions and in water availability that may become more important under stress, and with alterations in carbon allocation patterns, which may reflect the higher sensitivity of gymnosperms’ carbon economy to stress events (Adams et al., 2017). Some studies have shown stronger stomatal control and reduced non-structural carbohydrate (NSC) concentrations in tissues of dying conifers, relative to coexisting surviving individuals (Galiano et al., 2011; Timofeeva et al., 2017). For instance, *Pinus sylvestris* saplings survived experimental drought longer when keeping assimilation rates relatively high, even at the expense of higher water loss (Garcia-Forner et al., 2016). Associated changes in xylogenesis phenology are also likely to be important. Compared to healthy trees, defoliated pines showed a delay in the onset and reduction in the duration of cambial activity (Guada et al., 2016). Such physiological responses could explain the observed higher growth variability in dying trees that goes along with a different synchrony relative to surviving individuals.

In contrast, no consistent increase in growth variance was observed for angiosperms. This is in line with reported small and short-term reductions in tree growth before angiosperm death (Cailleret et al., 2017). Several reasons may explain the lack of growth-based signals in angiosperms, including greater functional diversity (Augusto et al., 2014), species-dependent responses to tree size compared to gymnosperms (Steppe et al., 2011), the relatively loose coupling between hydraulic failure and carbon depletion during drought (Adams et al., 2017), and their high recovery rates once favorable environmental conditions prevail after drought (Augusto et al., 2014; Anderegg et al., 2015b; Yin and Bauerle, 2017). Compared with gymnosperms, angiosperms generally have a higher capacity to (i) store NSC in their wood parenchyma (Plavcová et al., 2016), (ii) rebuild NSC pools owing to their higher stomatal conductance (Lin et al., 2015) and growth efficiency, and (iii) replace conducting area via new xylem growth (Brodribb et al., 2010), resprouting (Zeppel et al., 2015), and potentially by refilling embolized xylem conduits (Johnson et al., 2012). In addition, all gymnosperms studied are evergreen species, whereas most analyzed angiosperms are deciduous (except *Nothofagus betuloides, Nothofagus dombeyi*, and *Tamarix chinensis*) which may make them less dependent on previous-year leaf area and growth efficiency. The relatively low number of angiosperm species included in our study, together with the higher variation in leaf and growth strategies (e.g., diffuse- vs. ring-porous species) and in recovery performance across species relative to gymnosperms (Cailleret et al., 2017; Yin and Bauerle, 2017) may have also contributed to the lack of consistent increases in variance before tree mortality.

The lack of change in AR1 for both taxonomic groups may be explained by antagonistic effects of the stress-induced changes in key components of growth autocorrelation. On the one hand, the growth dependency on NSC reserves may induce lagged responses (‘growth memory’; Schulman, 1956; Esper et al., 2015; Timofeeva et al., 2017; von Arx et al., 2017). On the other hand, reductions in hydraulic conductivity through xylem embolism and lower production of new functional xylem (Brodribb et al., 2010), as well as reductions in overall crown area, or in leaf size, number and longevity (Bréda et al., 2006; Girard et al., 2012; Jump et al., 2017), may reduce the importance of lag effects. Finally, species-specific changes in water and carbon economy, during and after high stress levels (Galiano et al., 2017), can explain the lack of a consistent change in AR1 preceding tree death. For instance, after intense drought, carbon assimilates may be invested into storage and restoration of root functions rather than into stem growth (Palacio et al., 2012; Hagedorn et al., 2016; Martínez-Vilalta et al., 2016), and the allocation priority level varies among species (Galiano et al., 2017).

### Methodological Considerations

Our results did not agree with some previous studies that showed that declining/dying trees had higher radial growth variance, autocorrelation, and synchrony than healthy/surviving ones, or showed an increase of these growth metrics before death (e.g., Sánchez-Salguero et al., 2010; Amoroso et al., 2012; Camarero et al., 2015; Cailleret et al., 2016). They also indicate that the contrasting results obtained among previous studies (Supplementary Appendix A) may be due to methodological choices. In addition to the prescriptions that are inherent to the characteristics of our database, e.g., regarding the inequality in sample sizes among sites and species (Supplementary Appendix G), or the potential biases related to the assessment of the year of tree death (see Bigler and Rigling, 2013) or to the measurement of narrow rings, there are three particularly important elements to consider, which we discuss in the following paragraphs.

First, if one aims at understanding the ecological mechanisms behind changes in the variance (quantified here with SD) and autocorrelation of ring-width chronologies, the effects of tree size, growth level, and growth trend should be removed or accounted for. All these growth-related metrics are highly inter-correlated (Supplementary Appendix C), which can lead to a misinterpretation of the results. For instance, the decrease in SD_20_ calculated on raw RW data before tree death was caused by the gradual decrease in RW increment, and thus did not indicate an intrinsic decrease in growth sensitivity to inter-annual changes in environmental conditions (Figure 2A). Four procedures can be used to account for these effects: (i) detrending the RW time series to remove part of the low- and medium-frequency fluctuations, (ii) removing the remaining effects of growth rate on the composite SD, AR1 and COR individual time series using mixed-effects models, (iii) comparing dying and surviving trees with similar size or growth rate, and (iv) including the remaining differences in size and growth rate between dying and surviving trees of a given pair as an additional explanatory variable in the statistical models. As in all dendrochronological analyses, the detrending method should be carefully selected (e.g., Esper et al., 2015). For instance, the bandwidth of the kernel regression smoother should be constant among trees and should have an adequate length to capture enough medium-frequency (∼decadal-scale) variability (Supplementary Appendix D) while minimizing end-effect biases (Supplementary Appendix E). Also, and in contrast to classical dendroclimatic studies that aim at getting homoscedastic growth time series by calculating ratios (Cook and Peters, 1997; Frank et al., 2006), the heteroscedasticity of growth residuals needs to be retained. As using one or the other approach may lead to opposite trends (Supplementary Appendix C), differences are to be preferred over ratios (see also Scheffer et al., 2009; Dakos et al., 2012a).

Second, it is always advisable to combine both diachronic and synchronic approaches to control for potential biases that are typical of field data; i.e., to focus on the temporal change in growth metrics of dying trees before they actually die, and on the comparison between coexisting trees that died and survived a specific mortality event (see also Gessler et al., 2018). Still, the synchronic approach is prone to artifacts, due to the fact that the group of ‘surviving’ trees at a given mortality event, which are used as a control, may include trees that died shortly after the stress event. On the other hand, using the diachronic approach only is not sufficient to disentangle changes in growth patterns that are caused by variations in tree functions or in environmental conditions (e.g., mortality of neighbors). For instance, first-order temporal autocorrelation calculated for the 20-year period before the death of gymnosperms (AR1_20*f*_) was generally higher than average AR1_20_ (Figure 3B), which could indicate that high AR1 is associated with impending tree death. However, it cannot be used as a predictive tool, as high AR1 values were also observed during other periods of the trees’ lives, and because conspecific trees that survived the mortality event showed similar AR1_20*f*_ values (Figures 4C,D).

Third, the unexpected lack of significant and meaningful differences in growth-based EWS among the mortality groups considered here (see Cailleret et al., 2017) highlights the need for a more precise determination of the mortality source(s) in the field. It is now well accepted that tree mortality is a phenomenon induced by multiple biotic and abiotic drivers with strong interdependencies (Manion, 1991; Anderegg et al., 2015a), and rarely occurs because of one single factor. Trees in the ‘drought’ category might actually belong in ‘drought-biotic,’ and trees in the ‘others’ category might belong in the ‘biotic agents’ category (Das et al., 2016). In addition to information on climate, soil, and stand characteristics, detailed pathological data would be highly needed as biotic factors are involved in many individual mortality reports (Das et al., 2016).

### Application of Early-Warning Signals of Tree Mortality Based on Radial Growth

Our results expand previous assessments of the association between tree radial growth and mortality risk based on the direct effects of (absolute) growth rates (cf. Cailleret et al., 2017) by focusing on subtler properties of the growth time series. Overall, we found that an increase in inter-annual growth variability and a low growth synchrony could be used as EWS of gymnosperm mortality. Because these results were clear even after accounting for any indirect effect driven by changing growth levels, high growth variability and low synchrony could be used as independent diagnostics to identify gymnosperm trees or populations at high risk of mortality. However, these trends were much less consistent for angiosperms, and we did not find significant changes in autocorrelation prior to mortality. Hence, our results do not support the idea that critical slowing down indicators in radial growth data can be used as universal early warnings for tree mortality.

There are many reasons why early-warning indicators based on radial growth metrics may not be accurate indicators of stress-induced tree mortality. First, although we did not detect any consistent difference in growth metrics between mortality sources, some types of mortality stress may be too abrupt to be reflected in gradual changes in tree-ring width, and can occur without previous warning. For example, fungal diseases, bark-beetle outbreaks, or intense droughts can kill trees irrespective of their vitality, or at least, irrespective of their previous radial growth (Cherubini et al., 2002; Raffa et al., 2008; Sangüesa-Barreda et al., 2015; Cailleret et al., 2017). Second, for a similar stress event, there is a large variety in the type and timing of responses among and within species (Jump et al., 2017) that may confound detection of common changes in growth sensitivity. Third, annual radial growth may not be the most appropriate variable to derive such early warnings, as it is not only dependent on tree carbon and water status, but also on the environmental influences on sink activity (Körner, 2015). Other xylem-based physiological, anatomical, hydraulic, and isotopic properties that can be measured in tree rings may provide complementary information on tree mortality probability (e.g., Hereş et al., 2014; Anderegg et al., 2016a; Csank et al., 2016; Pellizzari et al., 2016; Timofeeva et al., 2017; Gessler et al., 2018). Fourth, despite recent developments (Gea-Izquierdo et al., 2015; Schiestl-Aalto et al., 2015; Guillemot et al., 2017), we lack mechanistic models of cambial activity based on sink demand, carbon uptake and reserves and water relations, which can go beyond simplistic formulations to produce clear expectations of ring-width dynamics before mortality (cf. Supplementary Appendix E). Finally, depending on which state variable(s) are affected by the environmental ‘noise’ and by the change in tree vitality, the temporal trends in AR1 and in SD prior to the transition can vary (Dakos et al., 2012b). For instance, the simple autoregressive models we developed to simulate decreasing growth rate over time, highlighted that all combinations of SD and AR1 trends can theoretically occur (Supplementary Appendix E). Considering that climate modifies tree growth based on multiple direct and indirect pathways (e.g., *via* changes in cambial activity and in the water and carbon economy), the relationship between climate variability and growth autocorrelation and variance is not straightforward. Similarly, the SD metric integrates both tree resistance and recovery to specific events that could be independently analyzed (Lloret et al., 2011; Dakos et al., 2015).

Climate change is predicted to modify mean temperature and precipitation, but also to increase the inter-annual variability and persistence of climatic fluctuations (Fischer et al., 2013; Lenton et al., 2017), and to modify the population dynamics of biotic agents (Allen et al., 2015). Several physiological thresholds can be exceeded during extreme biotic or abiotic conditions (e.g., during drought; Adams et al., 2017), which may ultimately lead to individual tree mortality, and potentially to widespread forest decline in many regions (Lloret et al., 2012; Reyer et al., 2013; Allen et al., 2015). However, we still lack a general set of mechanistic and empirical EWS of tree mortality at the individual scale (Gessler et al., 2018) that could be used to complement the signals used for detecting dieback at the forest stand or landscape scales (e.g., Verbesselt et al., 2016; Rogers et al., 2018). Based on a rich pan-continental ring-width database of dying and surviving trees, and by combining diachronic and synchronic approaches, our results highlight that in addition to the analysis of the multi-annual growth rates and trends (Cailleret et al., 2017), the inter-annual variability of the growth time series can be used to assess mortality risk, particularly for gymnosperm species.

## Author Contributions

MC, VD, and JM-V conceived the ideas and designed the methodology. MC, TA, MA, JA, CB, HB, J-JC, PC, MRC, KČ, AD, HD, GG-I, SG, LH, HH, A-MH, KH, PJ, JK, VK, TKi, TKl, TL, J-CL, FL, HM, IM, JM, WO, AP, AMP, BR, GS-B, JS, AS, DS, M-LS, MS, VT, RV, AW, PW, and JM-V collected the tree-ring data. MC, SJ, ER, and JM-V compiled and cleaned the ring-width database. MC analyzed the data and led the writing of the manuscript with inputs from VD and JM-V. All authors contributed critically to the drafts and gave final approval for publication.

## Conflict of Interest Statement

The authors declare that the research was conducted in the absence of any commercial or financial relationships that could be construed as a potential conflict of interest. The handling Editor declared a past co-authorship with the authors JC, PC, and KČ.
