# Supplementary material for: Early-Warning Signals of Individual Tree Mortality Based on Annual Radial Growth
Source: Front Plant Sci. 2019 Jan 8;9:1964. doi: 10.3389/fpls.2018.01964 (PMC6346433; doi:10.3389/fpls.2018.01964)
Supplement: Supplementary file 1 [file Data_Sheet_1.docx]

**Appendix A: Literature review of ring-width based early warning signals of tree mortality.**

**Table A1:** Literature review of several tree-ring based studies that compared the inter-annual variability and lag-1 autocorrelation (AR1) in growth between dying/declining and surviving/healthy trees (tree status as defined in the original manuscript) based on diachronic (diac) and/or synchronic (sync) analyses. Differences among these trees regarding Pearson correlation coefficients calculated between individual and site chronologies was also reported (COR). + and - mean that dying/declining trees had higher (orange color font) or lower value (blue color font) than surviving/healthy ones, respectively. = indicates no significant differences among both groups Different metrics were used to quantify the inter-annual variability in growth: Mean Sensitivity (MS), standard deviation (SD), variance (VAR), signal to noise ratio (SNR), coefficient of variation (CV), or drift-diffusion –jump metrics (DDJ). These metrics were calculated using different time windows (in brackets) from the entire tree-ring series (entire) to the *x* outermost rings.

| **Species** | **Article** | **Tree status** | **Type of analysis** | **Inter-annual variability** | **AR1** | **COR** |
| --- | --- | --- | --- | --- | --- | --- |
| *Abies alba* | Camarero et al. 2015 | declining | diac & sync | + (SD; 30) | + (30) | - (30) |
| *Abies alba* | Cailleret et al. 2016* | dying | diac & sync ∴ | + (SD; 10-50) | = (10-50) |  |
| *Abies cephalonica* | Papadopoulos et al. 2007 | dying | sync | + (SD; MS; entire) | + (entire) |  |
| *Abies concolor* | Kane and Kolb 2014 * | dying | sync | + (CV; 30-50) |  |  |
| *Austrocedrus chilensis* | Amoroso et al. 2012 * | declining/dying | sync | = (MS; entire) | + (entire) | + (entire) |
| *Fagus sylvatica* | Gillner et al. 2013 * | dying | sync | + (MS; 20) | = (5-30) |  |
| *Nothofagus dombeyi* | Suarez et al. 2004 * | dying | sync | + (MS; 18; 25) † |  |  |
| *Nothofagus dombeyi* | Cailleret et al. 2016* | dying | diac & sync ∴ | = (SD; 10-50) | = (10-50) |  |
| *Pinus albicaulis* | Millar et al. 2012 | dying | sync | - (RW vs climate; entire) |  |  |
| *Pinus banksiana* | Mamet et al. 2015 | dying | diac | = (DDJ) ‡ |  |  |
| *Pinus edulis* | Macalady and Bugmann 2014 | dying | sync | + (MS; 15) |  |  |
| *Pinus edulis* | Ogle et al. 2000 | dying | sync | + (VAR; 15) † |  |  |
| *Pinus flexilis* | Millar et al. 2007 | dying | diac & sync | + (SD; entire) |  |  |
| *Pinus flexilis* | Kane and Kolb 2014 * | dying | sync | + (CV; 5-10) |  |  |
| *Pinus halepensis* | Sanchez-Salguero et al. 2010 | declining | sync | + = (27)‡ | + = (27)‡ |  |
| *Pinus halepensis* | Camarero et al. 2015 | declining | diac & sync | + (SD; 30) | + (30) | + (30) |
| *Pinus nigra* | Herguido et al. 2016 | dying | sync | + = (MS; 58) ‡ |  |  |
| *Pinus sylvestris* | Gea-Izquierdo et al. 2014 * | dying | sync ∴ | + (MS; entire) | = (entire) |  |
| *Pinus sylvestris* | Herguido et al. 2016 | dying | sync | + (MS; 58) |  |  |
| *Pinus sylvestris* | Helama et al. 2012 | dying | sync | = (entire) | = (entire) |  |
| *Pinus sylvestris* | Timofeeva et al. 2017 | dying | sync | - (SD; 25 and entire) | + (25) |  |
| *Pinus sylvestris* | Camarero et al. 2015 | declining | diac & sync | = (SD; 30) | - (30) | + (30) |
| *Pinus pinaster* | Sanchez-Salguero et al. 2010 | declining | sync | = (27) | = (27) |  |
| *Pinus ponderosa* | Mc Dowell et al. 2010 | dying | sync | + (BAI vs PDSI; entire) |  |  |
| *Populus tremuloides* | Kane and Kolb 2014 * | dying | sync | + (CV; 30-50) |  |  |
| *Pseudotsuga menziesii* | Kane and Kolb 2014 * | dying | sync | + (CV; 10-50) |  |  |
| *Quercus petraea* | Cailleret et al. 2016* | dying | diac & sync ∴ | = (SD; 10-50) | = (10-50) |  |
| *Quercus pyrenaica* | Gea-Izquierdo et al. 2014 * | dying | sync ∴ | + (MS; entire) | = (entire) |  |
| *Quercus robur* | Helama et al. 2009 | declining | sync | + (MS; SD; entire) |  |  |
| *Quercus spp.* | Pedersen 1998 | dying | sync | - (linear model; entire) | = (entire) |  |

*indicates that the dataset was included in our database; † indicates that the difference was significant when focusing on the last 15 years while it was not significant when considering the entire tree-ring series; ‡ indicates significant differences among sites; ∴indicates that a logistic regression modeling approach has been used; depending on the statistical sampling design (focus only the year before mortality, or consider all growth observations), the approach is either synchronic, or diachronic and synchronic, respectively (details in Cailleret et al. 2016).

**References**

Amoroso, M. M., Daniels, L. D., & Larson, B. C. (2012). Temporal patterns of radial growth in declining Austrocedrus chilensis forests in Northern Patagonia: the use of tree-rings as an indicator of forest decline. *Forest ecology and management*, *265*, 62-70.

Cailleret, M., Bigler, C., Bugmann, H., Camarero, J. J., Cufar, K., Davi, H., ... & Martinez-Vilalta J. (2016). Towards a common methodology for developing logistic tree mortality models based on ring‐width data. *Ecological Applications*.

Camarero, J. J., Gazol, A., Sangüesa‐Barreda, G., Oliva, J., & Vicente‐Serrano, S. M. (2015). To die or not to die: early warnings of tree dieback in response to a severe drought. *Journal of Ecology*, *103*(1), 44-57.

Gea-Izquierdo, G., Viguera, B., Cabrera, M., & Cañellas, I. (2014). Drought induced decline could portend widespread pine mortality at the xeric ecotone in managed mediterranean pine-oak woodlands. *Forest Ecology and Management*, *320*, 70-82.

Gillner, S., Rüger, N., Roloff, A., & Berger, U. (2013). Low relative growth rates predict future mortality of common beech (Fagus sylvatica L.). *Forest ecology and management*, *302*, 372-378.

Helama, S., Läänelaid, A., Raisio, J., & Tuomenvirta, H. (2009). Oak decline in Helsinki portrayed by tree-rings, climate and soil data. *Plant and soil*, *319*(1-2), 163-174.

Helama, S., Läänelaid, A., Raisio, J., & Tuomenvirta, H. (2012). Mortality of urban pines in Helsinki explored using tree rings and climate records. *Trees*, *26*(2), 353-362.

Herguido, E., Granda, E., Benavides, R., García-Cervigón, A. I., Camarero, J. J., & Valladares, F. (2016). Contrasting growth and mortality responses to climate warming of two pine species in a continental Mediterranean ecosystem. *Forest Ecology and Management*, *363*, 149-158.

Kane, J. M., & Kolb, T. E. (2014). Short-and long-term growth characteristics associated with tree mortality in southwestern mixed-conifer forests. *Canadian Journal of Forest Research*, *44*(10), 1227-1235.

Macalady, A. K., & Bugmann, H. (2014). Growth-mortality relationships in piñon pine (Pinus edulis) during severe droughts of the past century: shifting processes in space and time. *PloS one*, *9*(5), e92770.

Mamet, S. D., Chun, K. P., Metsaranta, J. M., Barr, A. G., & Johnstone, J. F. (2015). Tree rings provide early warning signals of jack pine mortality across a moisture gradient in the southern boreal forest. *Environmental Research Letters*, *10*(8), 084021.

McDowell, N., Allen, C. D., & Marshall, L. (2010). Growth, carbon‐isotope discrimination, and drought‐associated mortality across a Pinus ponderosa elevational transect. *Global Change Biology*, *16*(1), 399-415.

Millar, C. I., Westfall, R. D., & Delany, D. L. (2007). Response of high-elevation limber pine (Pinus flexilis) to multiyear droughts and 20th-century warming, Sierra Nevada, California, USA. *Canadian Journal of Forest Research*, *37*(12), 2508-2520.

Millar, C. I., Westfall, R. D., Delany, D. L., Bokach, M. J., Flint, A. L., & Flint, L. E. (2012). Forest mortality in high-elevation whitebark pine (Pinus albicaulis) forests of eastern California, USA; influence of environmental context, bark beetles, climatic water deficit, and warming. *Canadian Journal of Forest Research*, *42*(4), 749-765.

Papadopoulos A, Raftoyannis Y, Pantera A (2007) Fir decline in Greece: a dendroclimatological approach. Proceedings of the 10th International Conference on Environmental Science and Technology. Kos Island, Greece.

Pedersen BS (1998) The role of stress in the mortality of midwestern oaks as indicated by growth prior to death. Ecology, 79, 79–93

Ogle, K., Whitham, T. G., & Cobb, N. S. (2000). Tree-ring variation in pinyon predicts likelihood of death following severe drought. *Ecology*, *81*(11), 3237-3243.

Sánchez-Salguero, R., Navarro, R. M., Camarero, J. J., & Fernández-Cancio, Á. (2010). Drought-induced growth decline of Aleppo and maritime pine forests in south-eastern Spain. *Forest Systems*, *19*(3), 458-470.

Suarez, M. L., Ghermandi, L., & Kitzberger, T. (2004). Factors predisposing episodic drought‐induced tree mortality in Nothofagus–site, climatic sensitivity and growth trends. *Journal of Ecology*, *92*(6), 954-966.

Timofeeva, G., Treydte, K., Bugmann, H., Rigling, A., Schaub, M., Siegwolf, R., & Saurer, M. (2017). Long-term effects of drought on tree-ring growth and carbon isotope variability in Scots pine in a dry environment. *Tree Physiology*, 1-14

**Appendix B: Characteristics of the tree-ring database**

**Table B1**: Detailed characteristics of the tree-ring datasets analyzed in the present study, including the number (nb) of dead trees considered, and of pairs of conspecific dying and surviving trees with similar diameter at breast height (DBH) or similar mean ring-width (RW) during the last 20 years (meanRW_20_). Trees with less than 20 rings were excluded from the dataset. When not measured in the original study, DBH was estimated as twice the sum of all previous ring-width measurements without considering bark thickness. The main sources of mortality were determined from field observations (presence/absence of biotic agents, stand structure) in combination with climatic analyses, and the following codes were used: D: Drought; B: Biotic agents that predispose (pred.; fungi, mistletoe, defoliator insects) or contribute (cont.; bark beetles, wood-borers) to tree mortality; Others (competition, frost or not Specified). As trees can die during the growing season and the precise (intra-annual) timing of tree death was not available, we did not consider the last ring of the dead trees. The year of death of the dead trees (period of mortality) was defined as the year of formation of the outermost ring, and considered as a proxy (cf. Bigler & Rigling, 2013). Ring-width data was available from cross-sections (C-sections) or from cores taken at different heights. At some sites, only the outermost rings of the cores were measured (Partial; in contrast to Complete).

| species | sites | article | Main mortality source | Nb dead trees | Nb pairs with similar  DBH / meanRW_20_ | Coring height (m) | Period of mortality | Data |
| --- | --- | --- | --- | --- | --- | --- | --- | --- |
| ***Abies alba*** Mill. | Bistra*  Ravnik  Issole2  Ventoux_TC  Ventoux_Dvx1  Ventoux_Dvx2  Ventoux_Dvx3  Vesubie3  Lopetón  Paco_Ezpela_High  Paco_Ezpela_Low  Canalicchio | Bigler et al. 2004  Bigler et al. 2004  Cailleret et al. 2014  Cailleret et al. 2014  Cailleret et al. 2014  Cailleret et al. 2014  Cailleret et al. 2014  Cailleret et al. 2014  Linares and Camarero 2012  Linares and Camarero 2012  Linares and Camarero 2012  Lombardi et al. 2008 | D  Others  D  DB (cont.)  DB (cont.)  DB (cont.)  DB (cont.)  D  D  D  D  Others | 2  14  8  67  8  11  10  9  2  3  3  47 | 0 / 0  12 / 6  8 / 4  67 / 64  7 / 3  10 / 8  9 / 4  8 / 5  0 / 0  2 / 2  3 / 2  44 / 27 | 1.3  1.3  1.3  1.3  1.3  1.3  1.3  1.3  1.3  1.3  1.3  1.3 | 1987  1988-1998  2003-2007  1998-2007  2004-2007  2003-2007  2002-2005  2003-2008  1996-1998  1996-1999  1998-2000  1955-1999 | Complete  Complete  Complete  Complete  Complete  Complete  Complete  Complete  Complete  Complete  Complete  Complete |
| ***Abies balsamea*** (L.) Mill. | Megantic  Amqui_1  Amqui_2  Chicoutimi_134  Chicoutimi_950  Degelis_1  Degelis_2  Depot_d’aigle_1  Foret_Montmorency_1  Lac_Gonzague_78  Lac_Humqui_1  Lac_Jacques_Cartier_79  Lac_Jacques_Cartier_2  LaMalbaie_970  LaMalbaie_145  New_Richmond_1  Parc_LaVerendrye | Filion et al. 1998  Coyea and Margolis 1994  Coyea and Margolis 1994  Coyea and Margolis 1994  Coyea and Margolis 1994  Coyea and Margolis 1994  Coyea and Margolis 1994  Coyea and Margolis 1994  Coyea and Margolis 1994  Coyea and Margolis 1994  Coyea and Margolis 1994  Coyea and Margolis 1994  Coyea and Margolis 1994  Coyea and Margolis 1994  Coyea and Margolis 1994  Coyea and Margolis 1994  Coyea and Margolis 1994 | B (pred.)  B (pred.)  B (pred.)  B (pred.)  B (pred.)  B (pred.)  B (pred.)  B (pred.)  B (pred.)  B (pred.)  B (pred.)  B (pred.)  B (pred.)  B (pred.)  B (pred.)  B (pred.)  B (pred.) | 43  5  1  6  5  5  7  1  7  3  6  1  1  8  7  5  9 | 43 / 42  5 / 1  1 / 0  6 / 2  5 / 3  4 / 1  7 / 4  1 / 1  7 / 5  3 / 2  6 / 3  1 / 0  1 / 1  8 / 2  7 / 4  5 / 2  7 / 1 | 0.3  1.3  1.3  1.3  1.3  1.3  1.3  1.3  1.3  1.3  1.3  1.3  1.3  1.3  1.3  1.3  1.3 | 1913-1987  1982-1988  1982  1974-1987  1979-1988  1979-1989  1977-1989  1989  1983-1987  1978-1989  1972-1989  1977-1986  1977-1985  1982-1988  1972-1985  1981-1989  1976-1989 | Complete  Complete  Complete  Complete  Complete  Complete  Complete  Complete  Complete  Complete  Complete  Complete  Complete  Complete  Complete  Complete  Complete |
| ***Abies cephalonica*** Loudon | Karpenissi  Agios_Nikolaos | Papadopoulos et al. 2007  Papadopoulos et al. 2007 | D  D | 10  5 | 8 / 7  5 / 4 | 1.3  1.3 | 2000-2003  2003 | Complete  Complete |
| ***Abies concolor*** (Gordon) Lindl. ex Hidebr. | LMCC  LogABCO  LogPILA  LogSEGI  AC_SUAB  AC_SUCR  AC_SUPI  AC_BWM  AC_SIT  AC_SFP | Das et al. 2007  Das et al. 2007  Das et al. 2007  Das et al. 2007  Das et al. 2007  Das et al. 2007  Das et al. 2007  Kane and Kolb 2014  Kane and Kolb 2014  Kane and Kolb 2014 | Others  Others  Others  Others Others Others Others  D  D  D | 40  16  17  47  28  35  17  25  2  1 | 39 / 30  13 / 7  8 / 8  45 / 40  23 / 16  28 / 26  14 / 12  23 / 16  2 / 0  1 / 0 | 1  1  1  1  1  1  1  1.3  1.3  1.3 | 1984-2001  1989-2001  1992-2001  1990-2001  1985-2001  1986-2001  1988-2001  1996-2007  1997-2004  2004 | Partial  Partial  Partial  Partial  Partial  Partial  Partial  Partial  Partial  Partial |
| ***Abies lasiocarpa*** (Hook.) Nutt | AL_TCRA  AL_TCLA  AL_TCRB  AL_TCLB  AL_CCLA*  AL_CCRA*  AL_CCRB  AL_PRLA  AL_STLB  AL_BCLA  AL_MPA  AL_ZLB  AL_ZLA  Adams_Lake  Damfino_Creek  Siccamous  AL_Col_M5* | Bigler et al. 2007  Bigler et al. 2007  Bigler et al. 2007  Bigler et al. 2007  Bigler et al. 2007  Bigler et al. 2007  Bigler et al. 2007  Bigler et al. 2007  Bigler et al. 2007  Bigler et al. 2007  Bigler et al. 2007  Bigler et al. 2007  Bigler et al. 2007  Antos et al. 2008  Antos et al. 2008  Antos et al. 2008  Smith et al. 2012 | D  D  D  D  D  D  D  D  D  D  D  D  D  Others  B (pred.)  B (cont.)  Others | 6  4  12  2  1  1  1  6  3  9  4  12  6  17  42  54  1 | 4 / 3  3 / 1  11 / 4  1 / 0  0 / 0  0 / 0  1 / 0  6 / 3  3 / 0  6 / 3  4 / 3  11 / 5  6 / 4  18 / 10  42 / 31  54 / 43  0 / 0 | 1.3  1.3  1.3  1.3  1.3  1.3  1.3  1.3  1.3  1.3  1.3  1.3  1.3  1.3  1.3  1.3  1.3 | 1920-2003  1944-1975  1937-2002  1988  1962  1959  1980  1935-1987  1955-1999  1947-1972  1940-2002  1922-2003  1947-2003  1936-1995  1875-1993  1925-1993  2005 | Complete  Complete  Complete  Complete  Complete  Complete  Complete  Complete  Complete  Complete  Complete  Complete  Complete  C-sections  C-sections  C-sections  Complete |
| ***Abies sibirica*** Ledeb. | East_Sayan | Kharuk et al. 2013 | DB (cont.) | 15 | 15 / 11 | 1.3 | 1990-2004 | C-sections |
| ***Acer saccharum*** Marshall | Temiscamingue | Hartmann et al. 2007 | B (pred.) | 53 | 53 / 51 | 1.3 | 1986-2004 | Complete |
| ***Austrocedrus chilensis*** (D.Don) Pic.Serm. & Bizzarri | CE1  CE2  CR1  CR2  EU1  EU2  K1  PP1  PP2  PP4  RQ1  RQ2  Confluencia  Centinela  Paso_del_viento | Amoroso et al. 2012  Amoroso et al. 2012  Amoroso et al. 2012  Amoroso et al. 2012  Amoroso et al. 2012  Amoroso et al. 2012  Amoroso et al. 2012  Amoroso et al. 2012  Amoroso et al. 2012  Amoroso et al. 2012  Amoroso et al. 2012  Amoroso et al. 2012  Villalba and Veblen 1998  Villalba and Veblen 1998  Villalba and Veblen 1998 | DB (pred.)  DB (pred.)  DB (pred.)  DB (pred.)  DB (pred.)  DB (pred.)  DB (pred.)  DB (pred.)  DB (pred.)  DB (pred.)  DB (pred.)  DB (pred.)  D  D  D | 13  14  8  11  17  13  18  15  44  7  29  18  27  6  6 | 13 / 7  13 / 7  8 / 6  10 / 7  17 / 6  12 / 7  16 / 9  14 / 9  44 / 33  7 / 3  29 / 17  17 / 9  26 / 14  5 / 1  5 / 1 | 0.3  0.3  0.3  0.3  0.3  0.3  0.3  0.3  0.3  0.3  0.3  0.3  1.3  1.3  1.3 | 1961-1999  1969-1995  1986-2005  1980-2001  1949-2003  1986-2000  1961-2000  1964-2003  1960-2002  1968-1996  1952-2001  1967-1999  1941-1957  1939-1958  1943-1959 | Complete  Complete  Complete  Complete  Complete  Complete  Complete  Complete  Complete  Complete  Complete  Complete  C-sections  C-sections  C-sections |
| ***Castanea sativa*** Mill. | Eisack_Valley | Waldboth and Oberhuber 2009 | DB (pred.) | 21 | 18 / 17 | 1.3 | 1970-2001 | Complete |
| ***Cupressus nootkatensis*** D.Don | BL_Prince_Rupert  HC_Prince_Rupert  SC_Prince_Rupert  WB_Prince_Rupert | Stan et al. 2011  Stan et al. 2011  Stan et al. 2011  Stan et al. 2011 | Others  Others  Others  Others | 14  18  11  7 | 14 / 9  17 / 15  11 / 11  6 / 7 | 0.3  0.3  0.3  0.3 | 1948-2001  1990-2005  1957-2001  1989-2002 | Complete  Complete  Complete  Complete |
| ***Fagus sylvatica*** L. | Borsberg  Montedimezzo | Gillner et al. 2013  Lombardi et al. 2008 | DB (pred.)  Others | 18  40 | 15 / 11  37 / 24 | 1.3  1.3 | 1982-2003  1947-1998 | Complete  Complete |
| ***Nothofagus betuloides*** (Mirb.) Oerst. | Navarino_Island | Lombardi et al. 2011 | Others | 34 | 33 / 21 | 1.3 | 1838-2005 | Complete |
| ***Nothofagus dombeyi*** (Mirb.) Oerst. | Cerro_Otto | Suarez et al. 2004 | D | 42 | 42 / 34 | 1.3 | 1998 | Complete |
| ***Picea abies*** (L.) H.Karst. | DP1  DP2  DP3  DP4  DP5  Boedmeren  Dischma  Fluehla  Scatle  Bystra  Hlinna  Medodoly  Pilsko  Ticha  Sipoo201  Sipoo202  Sipoo203  Sipoo204  Sipoo205  Hollola206  Hollola207  Hollola208  Merimasku  Askainen  Vahto  Paimio  Lammi | Aakala and Kuuluvainen 2011  Aakala and Kuuluvainen 2011  Aakala and Kuuluvainen 2011  Aakala and Kuuluvainen 2011  Aakala and Kuuluvainen 2011  Bigler and Bugmann 2004  Bigler and Bugmann 2004  Bigler and Bugmann 2004  Bigler and Bugmann 2004  Janda et al. 2017  Janda et al. 2017  Janda et al. 2017  Janda et al. 2017  Janda et al. 2017  Mäkinen et al. 2001  Mäkinen et al. 2001  Mäkinen et al. 2001  Mäkinen et al. 2001  Mäkinen et al. 2001  Mäkinen et al. 2001  Mäkinen et al. 2001  Mäkinen et al. 2001  Mäkinen et al. 2001  Mäkinen et al. 2001  Mäkinen et al. 2001  Mäkinen et al. 2001  Mäkinen et al. 2001 | DB (cont.)  DB (cont.)  DB (cont.)  DB (cont.)  DB (cont.)  Others Others  Others Others  B (cont.)  B (cont.)  B (cont.)  B (cont.)  B (cont.)  D  D  D  D  D  D  D  D  D  D  D  D  D | 16  13  13  8  12  9  13  17  8  14  45  33  15  35  6  5  8  4  14  2  3  3  12  8  7  3  11 | 16 / 8  13 / 8  13 / 7  6 / 3  11 / 5  7 / 3  12 / 9  16 / 8  6 / 5  11 / 8  43 / 43  33 / 24  14 / 10  33 / 32  6 / 5  4 / 4  8 / 4  4 / 2  14 / 9  2 / 2  3 / 1  3 / 1  12 / 10  8 / 4  7 / 5  3 / 1  11 / 10 | 1.3-6  1.3-6  1.3-6  1.3-6  1.3-6  1.3  1.3  1.3  1.3  1  1  1  1  1  1.3  1.3  1.3  1.3  1.3  1.3  1.3  1.3  1.3  1.3  1.3  1.3  1.3 | 1999-2003  2001-2006  2000-2005  2001-2005  1999-2007  1940-1994  1982-2000  1972-1998  1955-1997  2003-2010  2000-2012  2009-2012  2007-2012  2007-2011  1977-1997  1989-1997  1988-1997  1991-1995  1987-1997  1995  1992-1997  1994-1996  1983-1997  1997  1993-1997  1990-1994  1986-1996 | Complete  Complete  Complete  Complete  Complete  Complete  Complete  Complete  Complete  Complete  Complete  Complete  Complete  Complete  Complete  Complete  Complete  Complete  Complete  Complete  Complete  Complete  Complete  Complete  Complete  Complete  Complete |
| ***Picea engelmanii***  Parry ex Engelm. | PE_TCRA  PE_TCLA*  PE_TCLB  PE_CCLB  PE_CCRB  PE_CCRA  PE_PRLA  PE_PRLB  PE_STLB  PE_BCLA  PE_MPA  PE_MPB  PE_ZLB  PE_ZLA | Bigler et al. 2007  Bigler et al. 2007  Bigler et al. 2007  Bigler et al. 2007  Bigler et al. 2007  Bigler et al. 2007  Bigler et al. 2007  Bigler et al. 2007  Bigler et al. 2007  Bigler et al. 2007  Bigler et al. 2007  Bigler et al. 2007  Bigler et al. 2007  Bigler et al. 2007 | D  D  D  D  D  D  D  D  D  D  D  D  D  D | 3  2  2  4  1  1  3  5  1  5  8  3  1  9 | 1 / 1  0 / 1  2 / 0  1 / 3  1 / 0  1 / 0  1 / 0  5 / 2  1 / 0  4 / 2  7 / 3  2 / 0  1 / 0  7 / 2 | 1.3  1.3  1.3  1.3  1.3  1.3  1.3  1.3  1.3  1.3  1.3  1.3  1.3  1.3 | 1957  1953-1957  2000-2003  1962  1958  1963  1998  1928-1983  1979  1938-1996  1957-2002  1944-1960  1961  1976-1991 | Complete  Complete  Complete  Complete  Complete  Complete  Complete  Complete  Complete  Complete  Complete  Complete  Complete  Complete |
| ***Picea glauca*** (Moench) Voss | Site1_Tri  Site2_Oxy  Site3_Sib | Caccianiga et al. 2008  Caccianiga et al. 2008  Caccianiga et al. 2008 | B (cont.)  B (cont.)  B (cont.) | 21  18  19 | 19 / 11  18 / 4  15 / 6 | <0.3  <0.3  <0.3 | 1944-1997  1770-2001  1815-1995 | C-sections  C-sections  C-sections |
| ***Picea mariana*** (Mill.) Britton, Sterns & Poggenb. | D3  P3  D2  P2 | Westwood et al. 2012  Westwood et al. 2012  Westwood et al. 2012  Westwood et al. 2012 | B (pred.)  B (pred.)  B (pred.)  B (pred.) | 5  5  4  5 | 5 / 2  5 / 2  4 / 2  5 / 3 | 1.3  1.3  1.3  1.3 | 1996-2004  1998-2007  2003-2007  1983-2006 | Complete  Complete  Complete Complete |
| ***Pinus banksiana*** Lamb. | PMB  PSK  RMB  RSK | Metsaranta and Lieffers 2008  Metsaranta and Lieffers 2008  Metsaranta and Lieffers 2008  Metsaranta and Lieffers 2008 | Others  Others  Others  Others | 116  89  64  54 | 116 / 111  89 / 71  64 / 52  54 / 42 | 1.3  1.3  1.3  1.3 | 1957-2002  1933-2003  1955-2001  1943-2001 | Complete  Complete  Complete  Complete |
| ***Pinus brutia*** Ten. | Limnionas (Diss1) | Sarris unpub | D | 3 | 2 / 0 | 1.3 | 1999-2000 | Complete |
| ***Pinus contorta*** Douglas ex Loudon | PC_CCLB  PC_CCRA*  PC_STLB  PC_ZLA*  Col_M3  Col_M6  Col_P1  Col_P2 | Bigler et al. 2007  Bigler et al. 2007  Bigler et al. 2007  Bigler et al. 2007  Smith et al. 2012  Smith et al. 2012  Smith et al. 2012  Smith et al. 2012 | Others Others Others Others  B (cont.)  B (cont.)  B (cont.)  B (cont.) | 3  1  6  2  10  18  23  19 | 2 / 1  0 / 0  5 / 0  0 / 0  8 / 1  17 / 11  20 / 16  12 / 7 | 1.3  1.3  1.3  1.3  1.3  1.3  1.3  1.3 | 1973-1994  1962  1916-1977  1965-1969  1982-2008  1975-1994  1979-2005  1978-2005 | Complete  Complete  Complete  Complete  Complete  Complete Complete  Complete |
| ***Pinus flexilis*** E.James | PF_SFP  PF_SIT  Col_P3 | Kane and Kolb 2014  Kane and Kolb 2014  Smith et al. 2012 | D  D  D | 16  8  14 | 11 / 10  7 / 1  1 / 7 | 1.3  1.3  1.3 | 1996-2005  1997-2006  1975-2002 | Partial  Partial  Complete |
| ***Pinus halepensis*** Mill. | Yatir_forest  Lahav | Klein unpub  Dorman et al. 2015 | D  D | 11  20 | 11 / 7  19 / 13 | 1.3  1.3 | 2007-2008  2005-2010 | Complete  Complete |
| ***Pinus lambertiana*** Douglas | PL_SUAB  PL_SUCR  PL_SUPI | Das et al. 2007  Das et al. 2007  Das et al. 2007 | Others  Others  Others | 24  67  41 | 23 / 12  59 / 57  36 / 33 | 1  1  1 | 1976-2001  1982-2001  1987-2001 | Partial  Partial  Partial |
| ***Pinus mugo*** Turra | Swiss_National_Park_Engadine_Valley | Cherubini et al. 2002 | B (pred.) | 31 | 25 / 16 | 1 | 1973-1995 | Complete |
| ***Pinus ponderosa*** Douglas ex C.Lawson | Flagstaff_1  Flagstaff_139  Flagstaff_214  Flagstaff_243  Flagstaff_276  Flagstaff_278  Flagstaff_280 | Kane and Kolb 2010  Kane and Kolb 2010  Kane and Kolb 2010  Kane and Kolb 2010  Kane and Kolb 2010  Kane and Kolb 2010  Kane and Kolb 2010 | B (cont.)  B (cont.)  B (cont.)  B (cont.)  B (cont.)  B (cont.)  B (cont.) | 4  4  4  4  2  3  4 | 4 / 1  4 / 0  4 / 1  4 / 3  2 / 1  3 / 1  4 / 1 | 1.3  1.3  1.3  1.3  1.3  1.3  1.3 | 2000-2007  2003-2006  2003-2006  2001-2004  2002-2004  2005-2007  2001-2007 | Partial  Partial  Partial  Partial  Partial  Partial  Partial |
| ***Pinus sibirica*** Du Tour | BI2012 | Kharuk et al. 2013 | DB (cont.) | 19 | 18 / 14 | 1.3 | 1998-2010 | C-sections |
| ***Pinus sylvestris*** L. | Gliswald_Gamsen  Rohrberg_Eyholz  Valsain  Valsain_high  Arcalis_dry_transect  Prades_dry_transect  Tschirgant  Pfynwald  Solano_de_la_Vega_High  Solano_de_la_Vega_Low  Puerto_de_Gudar_Low  Puerto_de_Gudar_High | Bigler et al. 2006  Bigler et al. 2006  Gea-Izquierdo et al. 2014  Gea-Izquierdo et al. 2014  Hereş et al. 2012  Hereş et al. 2012  Oberhuber et al. 2001  Rohner unpub  Sangüesa-Barreda et al. 2013  Sangüesa-Barreda et al. 2013  Sangüesa-Barreda et al. 2013  Sangüesa-Barreda et al. 2013 | D  D  D  D  D  D  DB (pred.)  D  DB (pred.)  DB (pred.)  DB (pred.)  DB (pred.) | 20  23  17  16  25  14  57  53  2  3  3  5 | 20 / 16  23 / 20  15 / 8  16 / 10  24 / 18  11 / 6  57 / 39  53 / 44  1 / 0  2 / 3  3 / 1  4 / 4 | 1.3  1.3  1.3  1.3  1.3  1.3  1.3  1.3  1.3  1.3  1.3  1.3 | 1990-2001  1988-1998  1977-2012  1987-2011  1994-2007  1997-2007  1959-1996  1981-2006  1993  2001-2006  2003-2004  1994-2002 | Complete  Complete  Complete  Complete  Complete  Complete  Complete  Complete  Complete  Complete  Complete  Complete |
| ***Populus tremuloides*** Michx. | PT_BWM  PT_SIT  PT_SFP | Kane and Kolb 2014  Kane and Kolb 2014  Kane and Kolb 2014 | D  D  D | 7  4  49 | 7 / 3  4 / 0  46 / 37 | 1.3  1.3  1.3 | 1999-2007  2001-2005  1996-2007 | Partial  Partial  Partial |
| ***Pseudotsuga menziesii*** (Mirb.) Franco | PM_SIT  PM_SFP  PM_BWM | Kane and Kolb 2014  Kane and Kolb 2014  Kane and Kolb 2014 | D  D  D | 29  32  8 | 28 / 17  29 / 22  6 / 1 | 1.3  1.3  1.3 | 1998-2006  1996-2006  2001-2006 | Partial  Partial  Partial |
| ***Quercus cerris*** L. | Vojvodina | Stojanović et al. 2015 | D | 10 | 10 / 8 | 8 | 2012-2013 | C-sections |
| ***Quercus macrocarpa*** Michx. | Glacial_Lakes_State_Park  Maplewood_State_Park | Wyckoff and Bowers 2010  Wyckoff and Bowers 2010 | D  D | 11  4 | 8 / 6  4 / 1 | 0.3  0.3 | 2002  2002 | Partial  Partial |
| ***Quercus petraea*** (Matt.) Liebl. | Sikfokut  Runcu | Mészáros et al. unpub  Petritan et al. 2017 | Others  Others | 25  94 | 25 / 25  94 / 94 | 1.3  1.3 | 1954-2010  1965-2013 | Complete Complete |
| ***Quercus pyrenaica*** Willd. | QP_Valsain | Gea-Izquierdo et al. 2014 | D | 11 | 11 / 7 | 1.3 | 2003-2011 | Complete |
| ***Quercus robur*** L. | Cigonca  Chojnow_Forest_District | Levanic et al. 2011  Tulik 2014 | D  D | 5  5 | 1 / 2  2 / 0 | 4-5  1.3 | 2001  2008 | C-sections  C-sections |
| ***Quercus rubra*** L. | Red_Star-Ozarks  Mule_Farm-Ozarks  Ozarks-Cowell  Ozarks-Stack_Rock  Ouachitas-Dry_Creek_Mountain  Ouachitas-Flatside  Ouachitas-Talimena | Haavik et al. 2011  Haavik et al. 2011  Haavik et al. 2011  Haavik et al. 2011  Haavik et al. 2011  Haavik et al. 2011  Haavik et al. 2011 | DB (cont.)  DB (cont.)  DB (cont.)  DB (cont.)  DB (cont.)  DB (cont.)  DB (cont.) | 17  27  17  18  12  18  19 | 17 / 11  27 / 25  17 / 13  18 / 14  12 / 12  18 / 16  17 / 15 | 1.3  1.3  1.3  1.3  1.3  1.3  1.3 | 1977-2007  1991-2007  1979-2003  1978-2007  1989-2007  1989-2007  1996-2007 | Complete  Complete  Complete  Complete  Complete  Complete  Complete |
| ***Tamarix chinensis*** Lour. | Allen's_Patch  Army_Drain  Moab | Hultine et al. 2013  Hultine et al. 2013  Hultine et al. 2013 | B (pred.)  B (pred.)  B (pred.) | 1  6  5 | 0 / 0  6 / 2  4 / 1 | 1.5  1.5  1.5 | 2004-2009  2005-2008  2008-2010 | C-sections  C-sections  C-sections |

* indicates the sites that were not considered in Cailleret et al. (2017).

**References of the datasets already published**

Aakala T, Kuuluvainen T (2011) Summer droughts depress radial growth of *Picea abies* in pristine taiga of the Arkhangelsk province, northwestern Russia. Dendrochronologia, **29**, 67-75.

Amoroso MM, Daniels LD, Larson BC (2012) Temporal patterns of radial growth in declining *Austrocedrus chilensis* forests in Northern Patagonia: the use of tree-rings as an indicator of forest decline. Forest Ecology and Management, **265**, 62-70.

Antos JA, Parish R, Nigh GD (2008) Growth patterns prior to mortality of mature *Abies lasiocarpa* in old-growth subalpine forests of southern British Columbia. Forest Ecology and Management, **255**, 1568-1574.

Bigler C, Bräker O, Bugmann H, Dobbertin M, Rigling A (2006) Drought as an inciting mortality factor in Scots pine stands of the Valais, Switzerland. Ecosystems, **9**, 330-343.

Bigler C, Bugmann H (2004) Predicting the time of tree death using dendrochronological data. Ecological Applications, **14**, 902-914.

Bigler C, Gavin DG, Gunning C, Veblen TT (2007) Drought induces lagged tree mortality in a subalpine forest in the Rocky Mountains. Oikos, **116**, 1983-1994.

Bigler C, Gričar J, Bugmann H, Čufar K (2004) Growth patterns as indicators of impending tree death in silver fir. Forest Ecology and Management, **199**, 183-190.

Bigler C, Rigling A (2013) Precision and accuracy of tree-ring-based death dates of mountain pines in the Swiss National Park. Trees, **27**, 1703–1712.

Caccianiga M, Payette S, Filion L (2008) Biotic disturbance in expanding subarctic forests along the eastern coast of Hudson Bay. New Phytologist, **178**, 823-834.

Cailleret M, Nourtier M, Amm A, Durand-Gillmann M, Davi H (2014) Drought-induced decline and mortality of silver fir differ among three sites in Southern France. Annals of Forest Science, **71**, 643-657.

Cherubini P, Fontana G, Rigling A, Dobbertin M, Brang P, Innes JL (2002) Tree‐life history prior to death: two fungal root pathogens affect tree‐ring growth differently. Journal of Ecology, **90**, 839-850.

Coyea MR, Margolis HA (1994) The historical reconstruction of growth efficiency and its relationship to tree mortality in balsam fir ecosystems affected by spruce budworm. Canadian Journal of Forest Research, **24**, 2208-2221.

Das AJ, Battles JJ, Stephenson NL, Van Mantgem PJ (2007) The relationship between tree growth patterns and likelihood of mortality: a study of two tree species in the Sierra Nevada. Canadian Journal of Forest Research, **37**, 580-597.

Dorman M, Perevolotsky A, Sarris D, Svoray T (2015) The effect of rainfall and competition intensity on forest response to drought: lessons learned from a dry extreme. Oecologia, **177**, 1025-1038.

Filion L, Payette S, Delwaide A, Bhiry N (1998) Insect defoliators as major disturbance factors in the high-altitude balsam fir forest of Mount Mégantic, southern Quebec. Canadian Journal of Forest Research, **28**, 1832-1842.

Gea-Izquierdo G, Viguera B, Cabrera, M, Cañellas I (2014) Drought induced decline could portend widespread pine mortality at the xeric ecotone in managed mediterranean pine-oak woodlands. Forest Ecology and Management, **320**, 70-82.

Gillner S, Rüger N, Roloff A, Berger U (2013) Low relative growth rates predict future mortality of common beech (*Fagus sylvatica* L.). Forest Ecology and Management, **302**, 372-378.

Haavik LJ, Stahle DW, Stephen FM (2011) Temporal aspects of *Quercus rubra* decline and relationship to climate in the Ozark and Ouachita Mountains, Arkansas. Canadian Journal of Forest Research, **41**, 773-781.

Hartmann H, Messier C, Beaudet M (2007) Improving tree mortality models by accounting for environmental influences. Canadian Journal of Forest Research, **37**, 2106-2114.

Hereş AM, Martínez-Vilalta J, López BC (2012) Growth patterns in relation to drought-induced mortality at two Scots pine (*Pinus sylvestris* L.) sites in NE Iberian Peninsula. Trees, **26**, 621-630.

Hultine KR, Dudley TL, Leavitt SW (2013) Herbivory-induced mortality increases with radial growth in an invasive riparian phreatophyte. Annals of Botany, **111**, 1197-1206.

Janda P, Trotsiuk V, Mikoláš M, Bače R, Nagel TA, Seidl R, Seedre M, Morrissey RC, Kucbel S, Jaloviar P, Jasík M, Vysoký J, Šamonil P, Čada V, Mrhalová H, Lábusová J, Nováková M, Rydval M, Matějů L, Svoboda M (2017) The historical disturbance regime of mountain Norway spruce forests in the Western Carpathians and its inﬂuence on current forest structure and composition. Forest Ecology and Management, **388**, 67-78.

Kane JM, Kolb TE (2010) Importance of resin ducts in reducing ponderosa pine mortality from bark beetle attack. Oecologia, **164**, 601-609.

Kane JM, Kolb TE (2014) Short-and long-term growth characteristics associated with tree mortality in southwestern mixed-conifer forests. Canadian Journal of Forest Research, **44**, 1227-1235.

Kharuk VI, Im ST, Oskorbin PA, Petrov IA, Ranson KJ (2013) Siberian pine decline and mortality in southern Siberian Mountains. Forest Ecology and Management, **310**, 312-320.

Levanič T, Čater M, McDowell NG (2011) Associations between growth, wood anatomy, carbon isotope discrimination and mortality in a *Quercus robur* forest. Tree Physiology, **31**, 298-308.

Linares JC, Camarero JJ (2012) Growth patterns and sensitivity to climate predict silver fir decline in the Spanish Pyrenees. European Journal of Forest Research, **131**, 1001-1012.

Lombardi F, Cherubini P, Lasserre B, Tognetti R, Marchetti M (2008) Tree rings used to assess time since death of deadwood of different decay classes in beech and silver fir forests in the central Apennines (Molise, Italy*).* Canadian Journal of Forest Research, **38**, 821-833.

Lombardi F, Cocozza C, Lasserre B, Tognetti R, Marchetti M (2011) Dendrochronological assessment of the time since death of dead wood in an old growth Magellan's beech forest, Navarino Island (Chile). Austral Ecology, **36**, 329-340.

Mäkinen H, Nöjd P, Mielikäinen K (2001) Climatic signal in annual growth variation in damaged and healthy stands of Norway spruce [*Picea abies* (L.) Karst.] in southern Finland. Trees, **15**, 177-185.

Metsaranta JM, Lieffers VJ (2008) A fifty-year reconstruction of annual changes in the spatial distribution of *Pinus banksiana* stands: does pattern fit competition theory? Plant Ecology, **199**, 137-152.

Oberhuber W (2001) The role of climate in the mortality of Scots pine (*Pinus sylvestris* L.) exposed to soil dryness. Dendrochronologia, **19**, 45-55.

Papadopoulos A, Raftoyannis Y, Pantera A (2007) Fir decline in Greece: a dendroclimatological approach. Proceedings of the 10th International Conference on Environmental Science and Technology. Kos Island, Greece.

Petritan AM, Bouriaud O, Frank DC, Petritan IC. 2017. Dendroecological reconstruction of disturbance history of an old‐growth mixed sessile oak–beech forest. Journal of Vegetation Science, **28**, 117-127

Sangüesa-Barreda G, Linares JC, Camarero JJ (2013) Drought and mistletoe reduce growth and water-use efficiency of Scots pine. Forest Ecology and Management, **296**, 64-73.

Smith JM, Hart SJ, Chapman TB, Veblen TT, Schoennagel T (2012) Dendroecological reconstruction of 1980s mountain pine beetle outbreak in lodgepole pine forests in northwestern Colorado. Ecoscience, **19**, 113-126.

Stan AB, Maertens TB, Daniels LD, Zeglen S (2011) Reconstructing population dynamics of yellow-cedar in declining stands: baseline information from tree rings. Tree-Ring Research, **67**, 13-25.

Stojanović D, Levanič T, Matović B, Bravo-Oviedo A (2015) Climate change impact on a mixed lowland oak stand in Serbia. Annals of Silvicultural Research, **39**, 94-99.

Suarez ML, Ghermandi L, Kitzberger T (2004) Factors predisposing episodic drought‐induced tree mortality in *Nothofagus* – site, climatic sensitivity and growth trends. Journal of Ecology, **92**, 954-966.

Tulik M (2014) The anatomical traits of trunk wood and their relevance to oak (*Quercus robur* L.) vitality. European Journal of Forest Research, **133**, 845-855.

Villalba R, Veblen TT (1998) Influences of large-scale climatic variability on episodic tree mortality in northern Patagonia*.* Ecology, **79***,* 2624-2640.

Waldboth M, Oberhuber W (2009) Synergistic effect of drought and chestnut blight (*Cryphonectria parasitica*) on growth decline of European chestnut (*Castanea sativa*). Forest Pathology, **39**, 43-55.

Westwood AR, Conciatori F, Tardif JC, Knowles K (2012) Effects of *Armillaria* root disease on the growth of *Picea mariana* trees in the boreal plains of central Canada. Forest Ecology and Management, **266**, 1-10.

Wyckoff PH, Bowers R (2010) Response of the prairie–forest border to climate change: impacts of increasing drought may be mitigated by increasing CO2. Journal of Ecology, **98**, 197-208.

**Table B2**: Main characteristics of the tree-ring width database described in Table B1, showing details about the number of species and sites studied, the number of dying trees, and the number of pairs of conspecific dying and surviving trees with similar diameter at breast height (DBH) or similar mean ring-width (RW) during the 20-years period before tree death, by group of mortality source.

|  | | **Drought** | **Drought + Biotic** | **Biotic agents** | **Others** |
| --- | --- | --- | --- | --- | --- |
| **species** | angiosperms | 6 | 3 | 2 | 3 |
|  | gymnosperms | 12 | 6 | 8 | 8 |
| **sites** | angiosperms | 10 | 9 | 4 | 4 |
|  | gymnosperms | 70 | 28 | 43 | 30 |
| **dying trees** | angiosperms | 148 | 167 | 65 | 193 |
|  | gymnosperms | 619 | 469 | 561 | 843 |
| **Pairs with similar DBH** | angiosperms | 135 | 160 | 66 | 191 |
|  | gymnosperms | 561 | 455 | 531 | 788 |
| **Pairs with similar mean RW_20_** | angiosperms | 98 | 134 | 54 | 165 |
|  | gymnosperms | 354 | 302 | 354 | 631 |

**Appendix C: Effect of the RW detrending method on the calculation of SD and AR1**


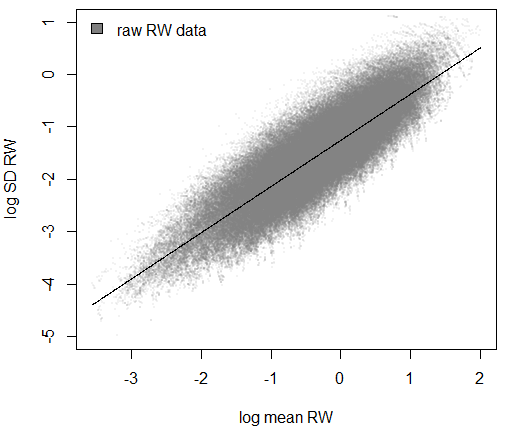

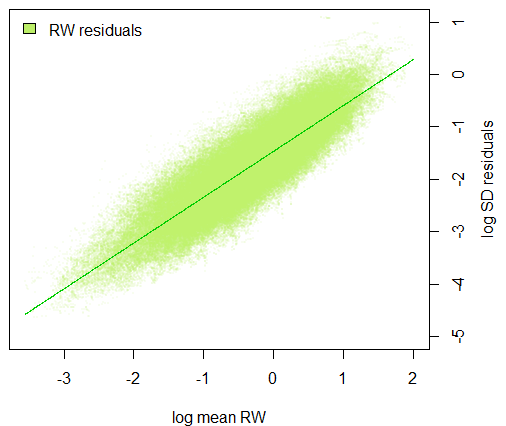


**(b)**

**(a)**

**(d)**


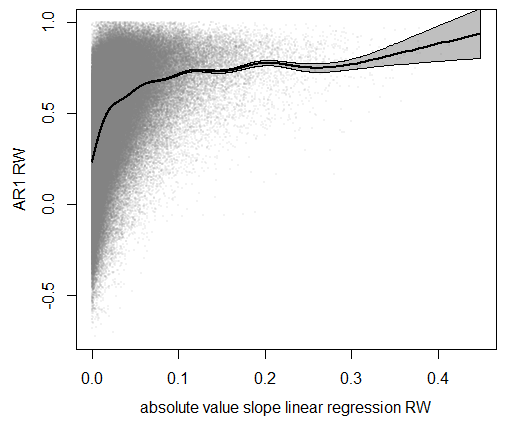

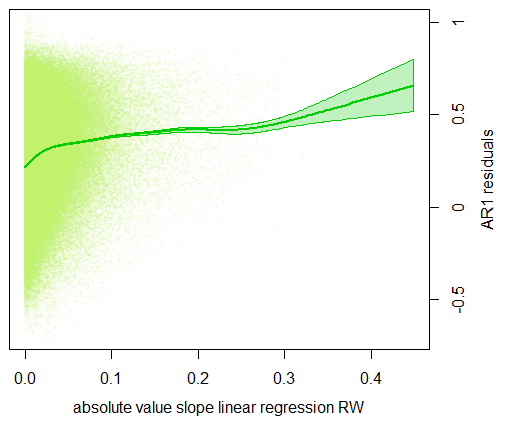


**(c)**


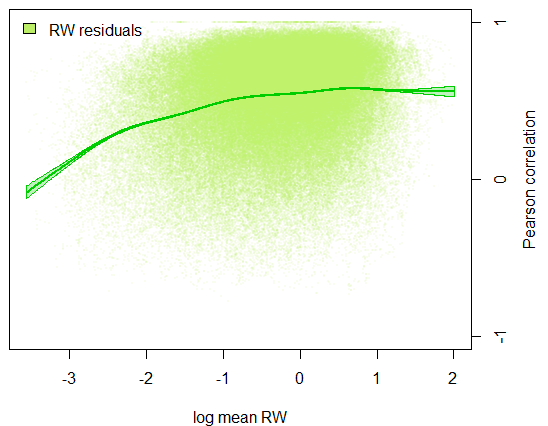


**(e)**

**Figure C1**: Effect of the detrending on the relationship between mean RW and SD calculated on the original (a; black) and on detrended RW data (b; green), and on the relationship between growth trend (slope of the linear regression fitted to raw RW data) and AR1 calculated on the original (c) and detrended RW data (d). (e) Relationship between mean RW and Pearson correlation coefficient. Both SD and mean RW were log-transformed. Each variable was calculated using a 20-years window. Only data from dead trees were shown here (all observations were used), but results were similar with surviving ones. Relationships were represented using general additive models (lines and shaded areas).


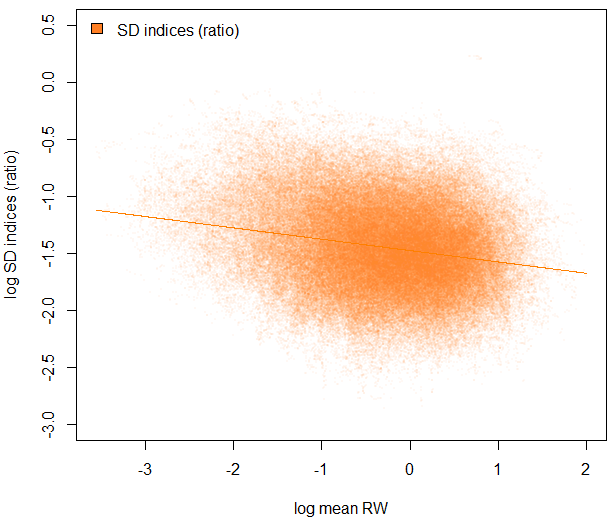

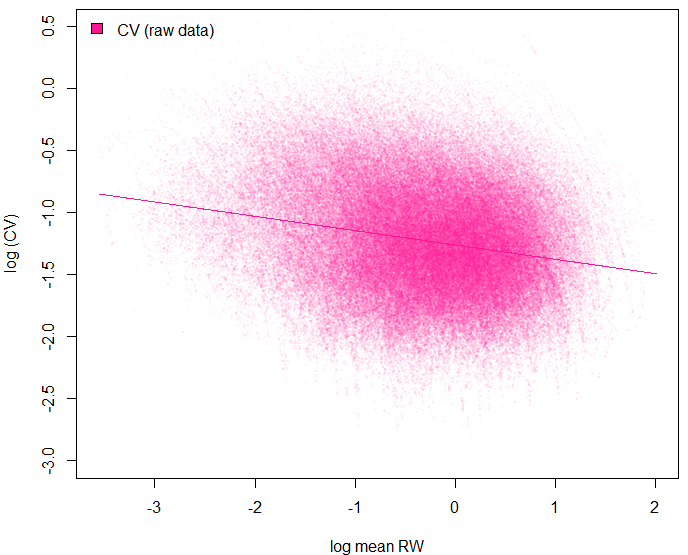

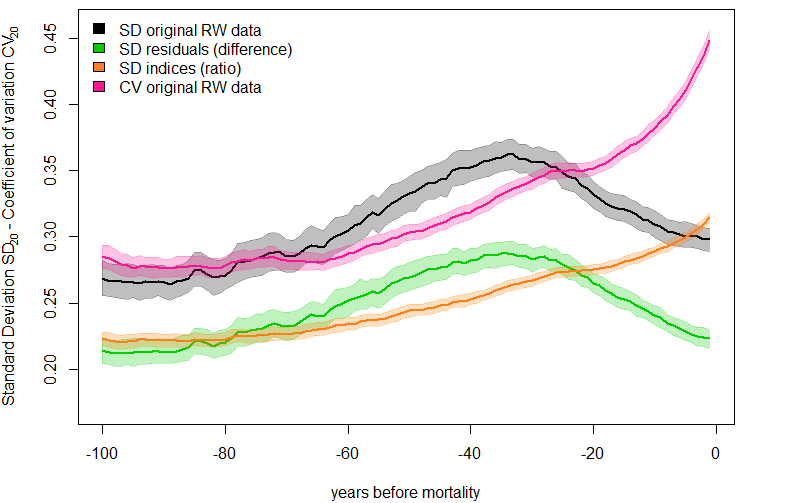


**(c)**

**(b)**

**(a)**

**Figure C2**: (a) Relationship between log-transformed mean RW and log-transformed SD calculated on RW indices (ratio between raw RW data and the RW predicted by the Gaussian filtering with a 15-year bandwidth; calculated for dead trees only). (b) Relationship between log-transformed mean RW and log-transformed CV (SD/RW) calculated on the original RW data. (c) Temporal change before tree death in SD calculated on raw RW data (black), on RW residuals (difference; green) and on RW indices (ratio; orange), and in CV calculated on raw RW data (pink). Each growth variable was calculated with a 20-year moving time-window.

Even if time-series of growth ratios should be homoscedastic (Cook 1987^[[1]](#footnote-1)^), their variance is still dependent on the initial growth rate, with a general negative relationship (Fig. C2a). In consequence, the constant increase in SD_20_ observed for dying trees before their death (Fig. C2c) cannot be fully attributed to intrinsic changes in their growth variability. Similar interpretation applies to CV_20_ (Fig. C2a and C2c).**Appendix D: Effect of the bandwidth of the kernel regression smoother to detrend RW time-series**


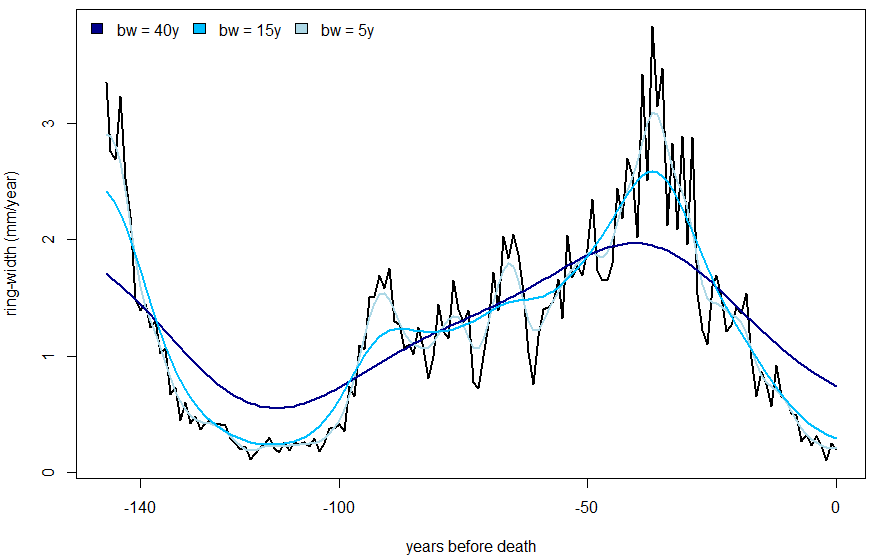

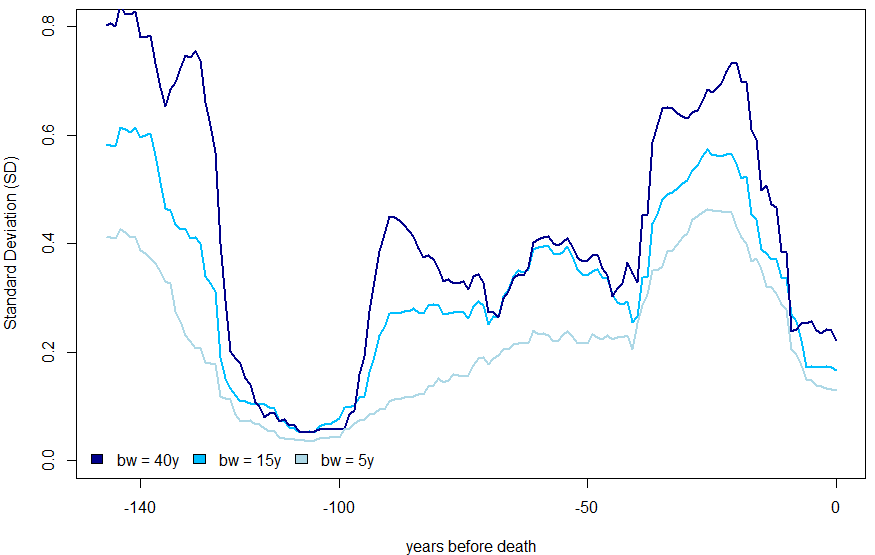

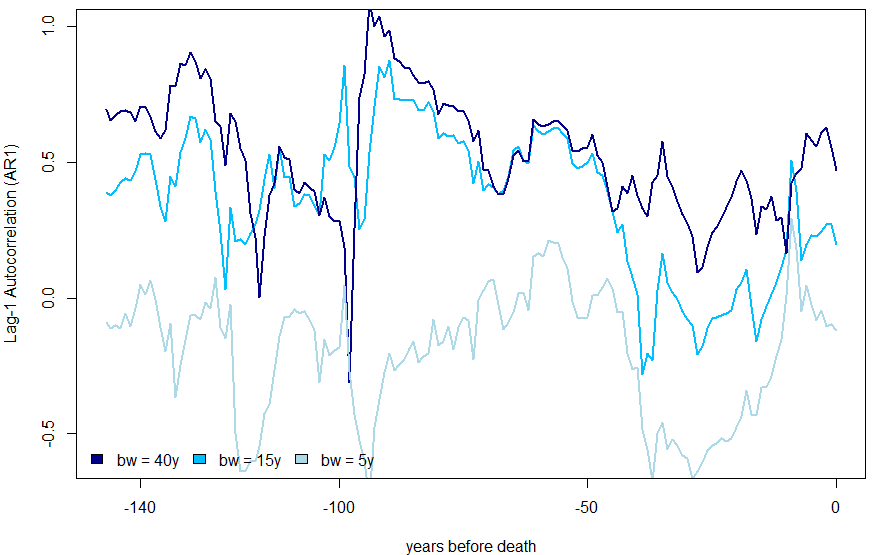


**Figure D1:** Impact of the use of different bandwidths (bw) of the kernel regression smoother to detrend raw RW time-series (in black) on the level and temporal change in AR1 and SD calculated on the detrended data for the tree “01069” from the site “Ravnik” (*Abies alba*; Bigler et al. 2004).


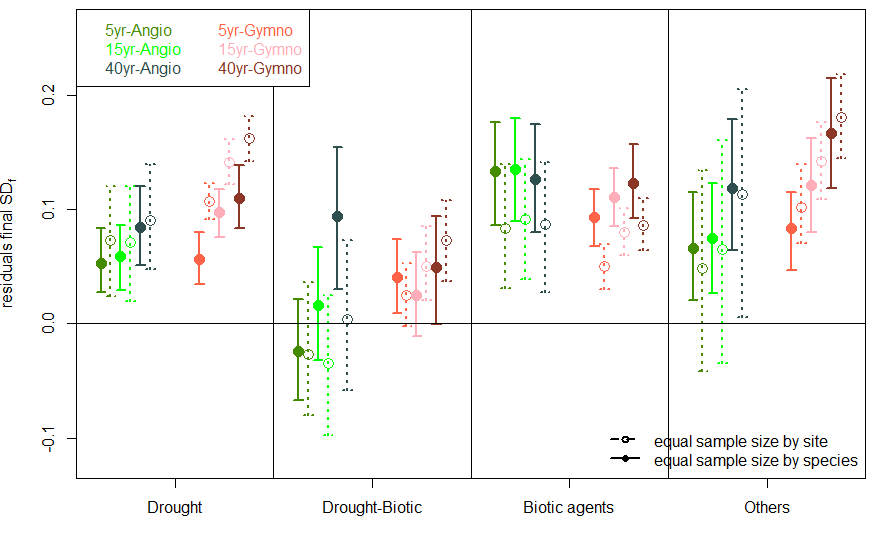

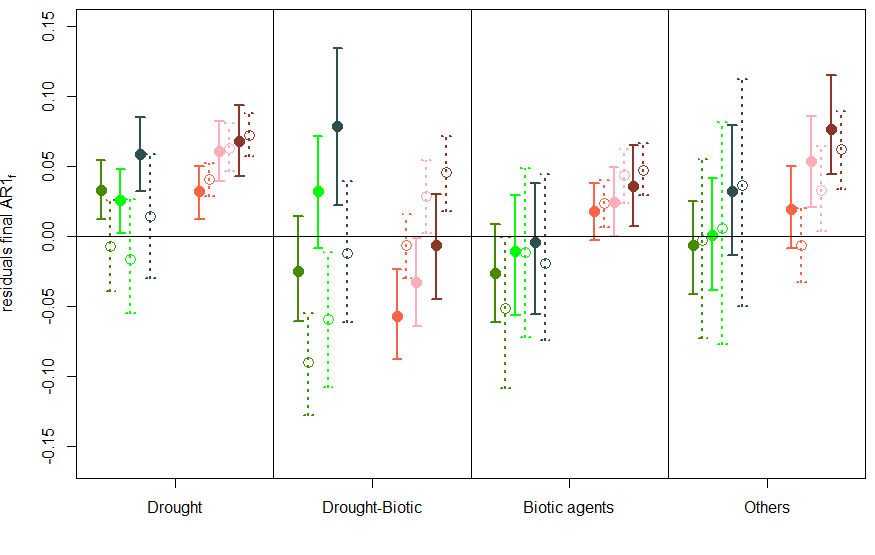

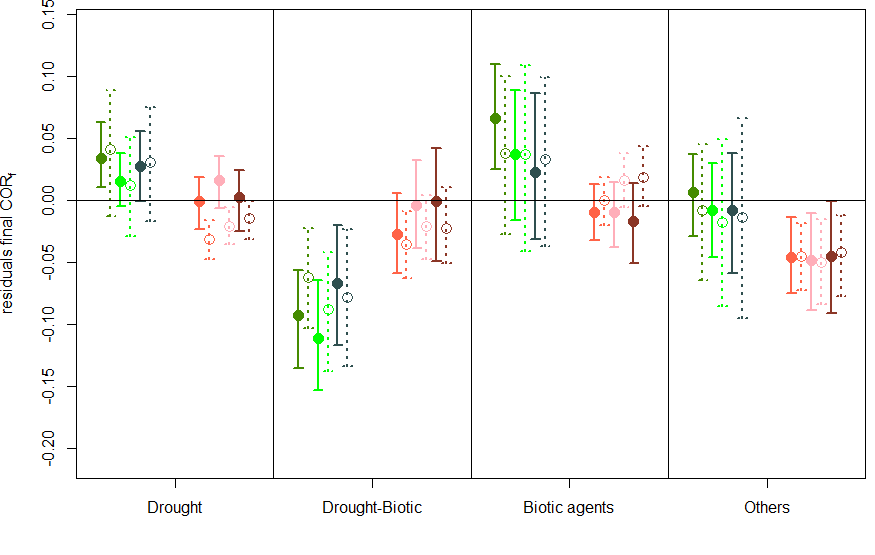


**Figure D2**: Variation in the residuals of final SD_20_, AR1_20_, and COR_20_ of dying trees according to the length of the bandwidth of the kernel regression smoother used to detrend RW data. Three fixed bandwidths were tested: 5-years, 15-years (corresponds to the data shown in the main text; Figure 3), and 40-years. Data were shown for both angiosperms (greenish color) and gymnosperms (reddish color) and for the two re-sampling strategies (detailed in Appendix F).

On the one hand, splines with large bandwidths (i.e., 40 years) do not capture enough medium frequency variability (Fig. D1), and may induce strong ‘end-effects’ biases (Appendix E). SD values calculated on the residuals obtained with these splines are higher than SD values obtained for a bandwidth <20 years, especially when growth rates are rapidly changing (e.g., from -145 to -130 years in Fig. C1).

On the other hand, using very short bandwidths (e.g., 5 years) reduces ‘end-effects’ biases (Appendix E) but may capture too much high-frequency variability and autocorrelation. In consequence, the corresponding SD and AR1 values are quite low (Fig. D1)

Nevertheless, using one or another bandwidth does not significantly impact the analysis of the residuals of final SD_20_, AR1_20_, and COR_20_ of dying trees (Fig. D2).

**Reference:**

Bigler C, Gričar J, Bugmann H, Čufar K (2004) Growth patterns as indicators of impending tree death in silver fir. Forest Ecology and Management, **199**, 183-190.

**Appendix E: Expected EWS based on theoretical autoregressive models**

Considering that mortality is commonly preceded by a long-term decrease in growth (Cailleret et al. 2017), we aim at generating ring-width time-series with constant growth (reference, hereafter called RW series of ‘surviving trees’) and with a gradual decrease in growth (for ‘dying trees’).

To mimic RW time-series, we built 2 simple growth models that include (i) an autocorrelation component (*α*), (ii) a long-term change in the mean (*μ*), and (iii) some noise reflecting the environmental stochasticity (*ε*).

${RW}_{t+1}=max\left( 0,\alpha_{t}{*RW}_{t}+\mu_{t}+\varepsilon_{t} \right)$ [model 1]

${RW}_{t+1}={(\alpha}_{t}+\varepsilon_{t}){RW}_{t}+\mu_{t}$ [model 2]

with

{*μ_t_* = *μ_0_*; *α_t_* = *α_0_*} for surviving trees;

{*μ_t_* = *μ_0_* - *μ_A_*; *α_t_* = *α_0_*} for dying trees A;

{*μ_t_* = *μ_0_*; *α_t_* = *α_0_*  - *α_B_*} for dying trees B.

{*μ_t_* = *μ_0_* – *μ_C_*; *α_t_* = *α_0_*  + *α_C_*} for dying trees C.

For surviving trees, RW of the next year (*t+1*) depends on the current RW (*t*) with constant rate *α_o_*, and on a constant “background growth” *μ_0_*. For dying trees A, the background growth is gradually decreasing (*μ_t_* = *μ_o_* - *μ_A_*) while the autocorrelation component stays constant (*α_t_* = *α_0_*). For dying trees B, the autocorrelation component is gradually decreasing (*α_t_* = *α_0_*  - *α_B_*), and the background growth constant over time (*μ_t_* = *μ_o_*). For dying trees C, the background growth is gradually decreasing (*μ_t_* = *μ_o_* – *μ_C_*), but the autocorrelation component increases over time (*α_t_* = *α_0_* + *α_C_*).

RW of dying trees decreases over time either due to a reduction in long-term mean (*μ*; dying trees A and C; can lead to a zero RW) or in the autocorrelation component (*α*; dying tree B; RW reaches an equilibrium at RW_1_/(1- *α_o_*); see Fig. E1).

The magnitude of the effect of the environmental stochasticity (noise *ε_t_*) is either independent (model 1) or dependent (model 2) on tree RW. Also, it is either constant, or increases over time (irrespective of tree RW).

**Simulated ring-width chronologies for surviving and dying trees**

200 RW chronologies of 100 years were generated for the surviving and dying trees (A, B, and C), and we calculated the corresponding relative growth rates (RW_t+1_/RW_t_), SD and AR1 of the detrended chronologies in moving windows of 20 years. Two Gaussian smoothers with different bandwidths (5 and 20 years) were tested to assess potential ‘end effects’ biases due to inaccurate detrending of RW chronologies at both edges. In this example we assume that surviving and dying trees experience the same environmental stochasticity (same noise *ε_t_*). The following parameter values were used:

*μ_0_* = 0.3; *μ_A_* = *μ_C_* = 0.003; *α_0_* = 0.7; *α_B_* = 0.007; *α_C_* = 0.0025

*ε_t_* = N(mean=0, sd=0.005+*ε_0_*) for model 1; *ε_t_* = N(mean=0, sd=0.05+*ε_0_*) for model 2; with *ε_0_*=0 or *ε_0_*=0.004**t* if the environmental noise is constant or increases over time (*t*), respectively.

**Results**

RW of surviving trees fluctuate around 1 mm/year, while RW of dying trees decreases over time following three types of functions: linear (dying tree A; linear decrease in the background growth μ), negative exponential (dying tree B; linear decrease in the autocorrelation component α), and ‘pseudo-logarithmic’ (linear decrease in the background growth μ combined with a linear increase in the autocorrelation component α; Figs. E1 to E5). We could thus mimic 3 types of growth decline patterns: (i) linear, (ii) decreasing rate of growth decline before mortality, (iii) increasing rate of growth decline before mortality.

Relative growth rates gradually decline for the dying trees A and C (with the decrease in the background growth μ), but stay constant for dying trees B (Figs. E2, E3).

The analysis of SD and AR1 of dying trees highlights the importance of the choice of the bandwidth of the Gaussian smoother. With a 20-year bandwidth, strong ‘end-effects’ can be observed (e.g., Fig. E2) because the smoother doesn’t accurately fit the RW data at both edges of the chronology (Fig. E1). With a shorter bandwidth (e.g., 5 years) SD and AR1 values are lower, but ‘end-effects’ are less present (e.g., Fig. E2).

Regardless of this effect, the change in SD over time for dying trees is highly dependent on the model features:

- Stable when the noise *ε_t_* is constant and is independent of tree RW (model 1; Fig. E2).
- Increase when the noise *ε_t_* increases over time and is independent of tree RW (model 1; Fig. E4).
- Decreases when the noise *ε_t_* is constant but depends on tree RW (model 2; Fig. E3).
- All temporal trends can be obtained when the environmental noise *ε_t_* increases over time and depends on tree RW (model 2; Fig. E5).

For AR1, it decreases over time for dying trees B, is stable for dying trees A, and increases for dying trees C, irrespective of the model and of the noise used.

To conclude, all possible combinations in the temporal trends of SD and AR1 can be obtained with the decrease in tree growth. It depends on the shape and nature of the decrease in RW over time, and on the change in the environmental noise.


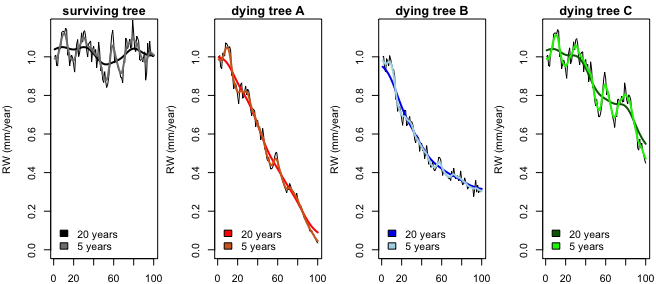


**Figure E1**: Example of RW time-series simulated using model 2 with constant environmental noise for a surviving tree, and dying trees A, B, and C (from *Left* to *Right*). Detrended RW data with Gaussian smoothers (5- and 20-years bandwidths) are also indicated.

**
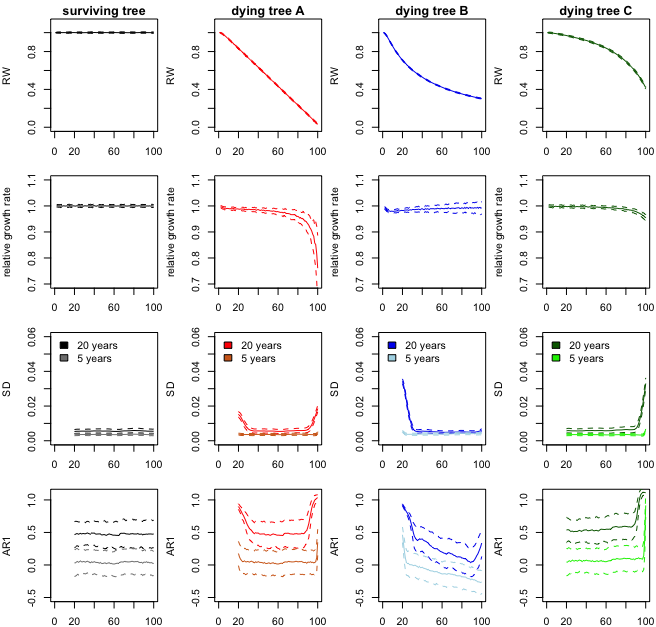
**

**Figure E2.** Average temporal change in simulated RW, relative growth rate, Standard Deviation (SD) and lag-1 autocorrelation (AR1) simulated using the model 1 without temporal change in the environmental noise. RW data were detrended using Gaussian smoothers with 5- and 20-year bandwidths.


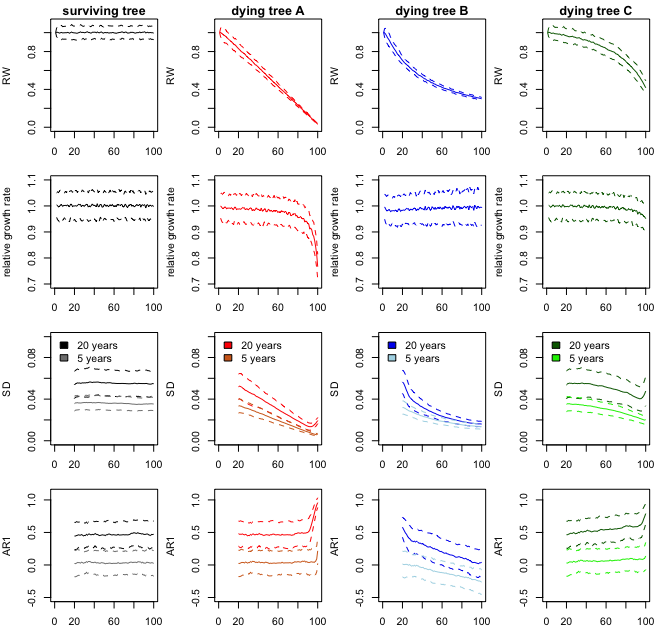


**Figure E3.** Average temporal change in simulated RW, relative growth rate, Standard Deviation (SD) and lag-1 autocorrelation (AR1) simulated using the model 2 without temporal change in the environmental noise. RW data were detrended using Gaussian smoothers with 5- and 20-year bandwidths.

**
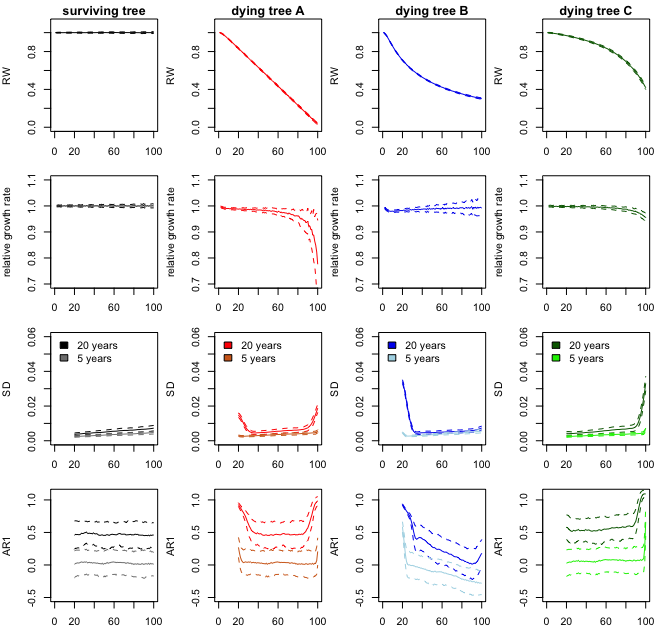
**

**Figure E4**. Average temporal change in simulated RW, relative growth rate, Standard Deviation (SD) and lag-1 autocorrelation (AR1) simulated using the model 1 with temporal increase in the environmental noise. RW data were detrended using Gaussian smoothers with 5- and 20-year bandwidths.

**
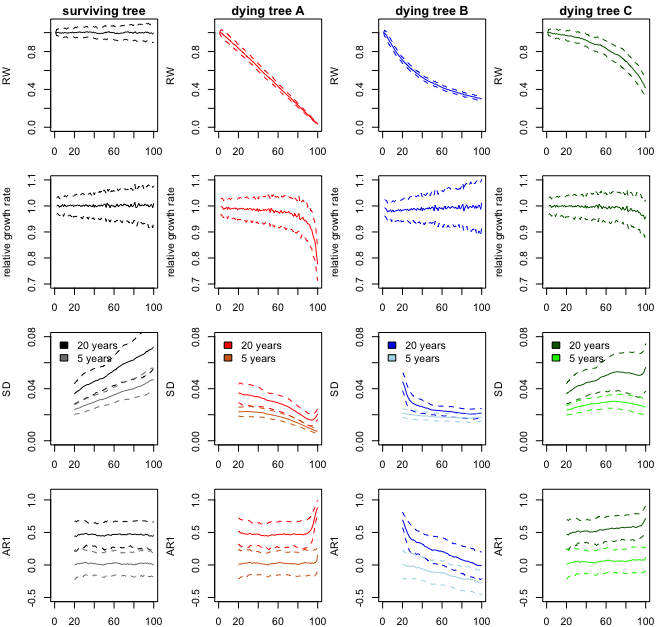
**

**Figure E5**. Average temporal change in simulated RW, relative growth rate, Standard Deviation (SD) and lag-1 autocorrelation (AR1) simulated using the model 2 with temporal increase in the environmental noise.

**Appendix F: Effect of the length of the moving time-window on SD, AR1, and COR.**


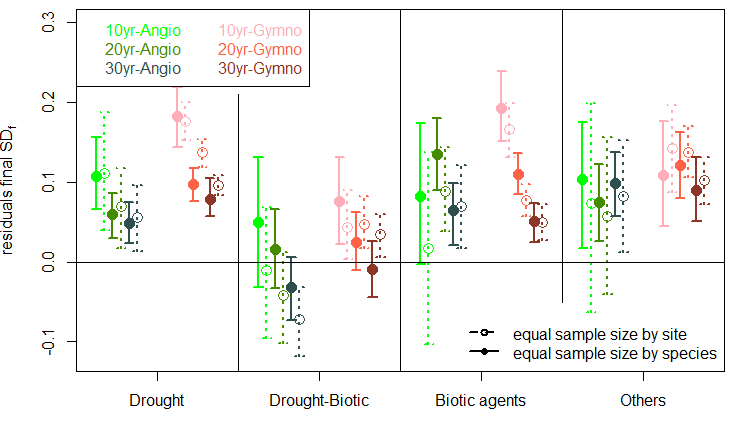

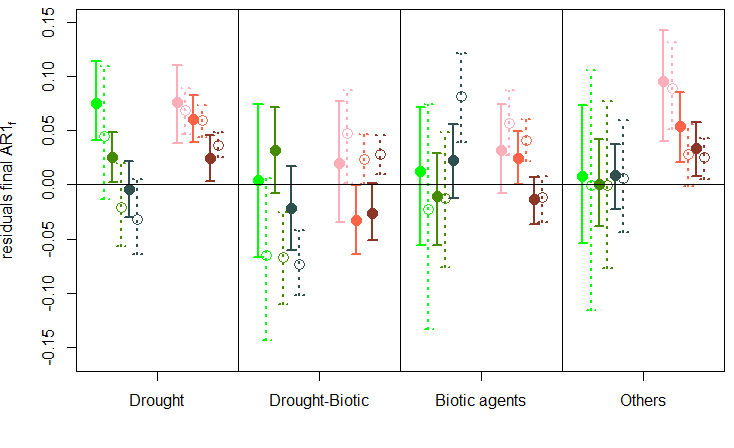

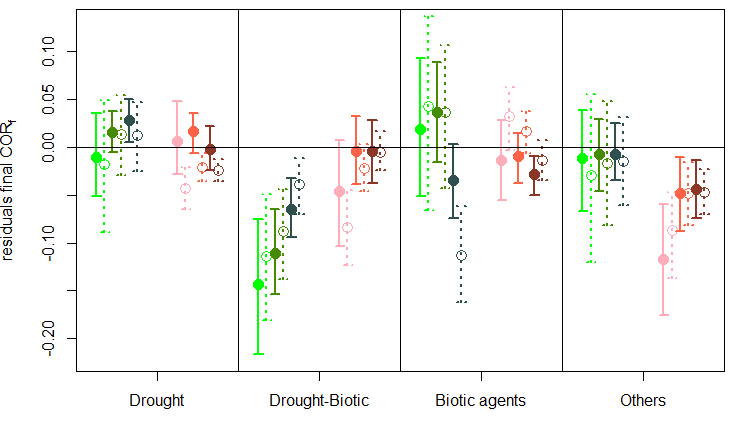


**Figure F1**: Variation in the residuals of final SD, AR1, and COR of dying trees according to the length of the moving time-window used. Three windows were tested: 10-years, 20-years (corresponds to the data shown in the main text; Figure 3) and 30-years. Data were shown for both angiosperms (greenish color) and gymnosperms (reddish color) and for the two re-sampling strategies (detailed in Appendix G).

**Appendix G: Resampling procedures to consider for the heterogeneity in sampling properties**

To account for the heterogeneity in the number of dying trees per site and per species in the dataset, we used two resampling procedures (Cailleret et al. 2017).

*Diachronic approach*

In the first resampling procedure, we randomly sampled with replacement 53 dying trees (median of the number of dying trees per species in the database; 1908 chronologies per dataset) for each of the 36 species. Depending on the species, the information from a given mortality event could be either replicated or excluded. This sampling procedure was repeated 500 times and averaged resSD_20_, resAR1_20_, and resCOR_20_ values were calculated for each of these 500 datasets. In the second resampling procedure, we generated 500 datasets for which we randomly sampled with replacement 10 dying trees for each of the 199 sites (median in the database; 1990 chronologies per dataset).

*Synchronic approach*

As before, we used species-specific and site-specific resampling procedures to account for the differences in sampling intensity between species and sites in the LMM analyses. For the species-specific resampling, we generated 500 different datasets by randomly sampling with replacement 47 or 34 mortality events (~pair dying-surviving) per species (medians in the database when dying and surviving trees from a given pair had similar DBH_f_ or similar meanRW_20f_, respectively). In the case of the site-specific resampling we generated 500 different datasets by randomly sampling with replacement 8 or 7 mortality events per site.

With both approaches, each species or each site has the same weight in the calibration dataset and contributes equally to the results. Nonetheless, sampling a similar number of trees per species may overemphasize the effect of species with low initial number of sample trees and sites (e.g., *Pinus brutia*). At the same time, sampling a similar number of trees per sites may overemphasize species with high number of sample sites (e.g., *Picea abies*). Thus, combining both resampling methods aimed at minimizing the bias related to under-sampling or over-sampling for specific sites or species.


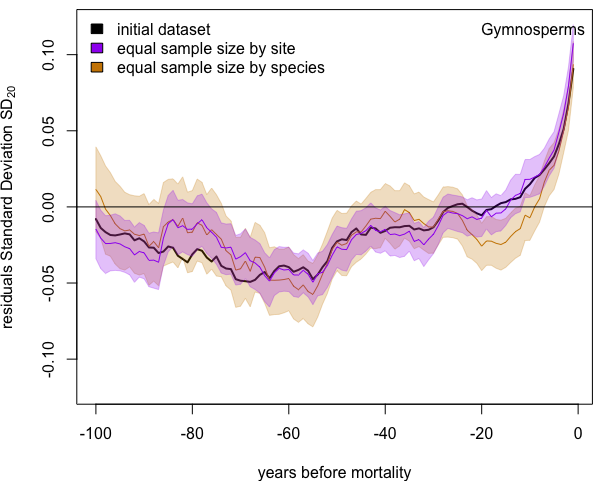

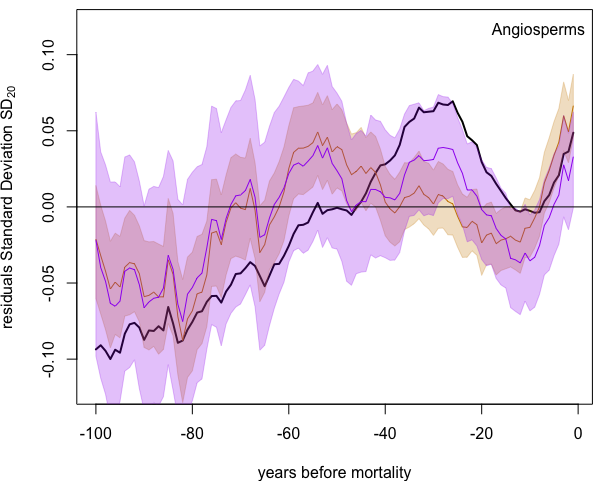


**(b)**

**(a)**

**
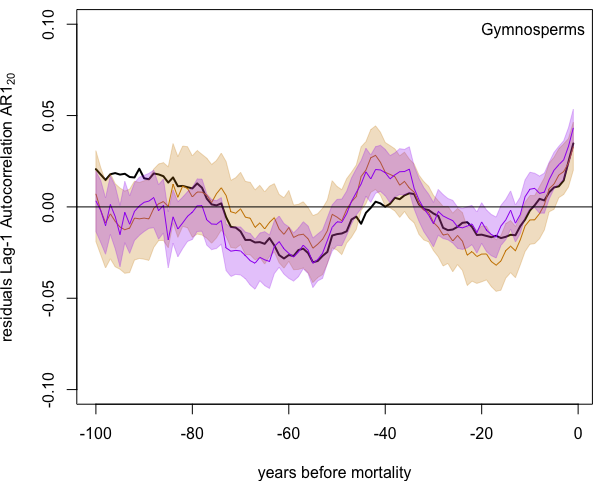
** **
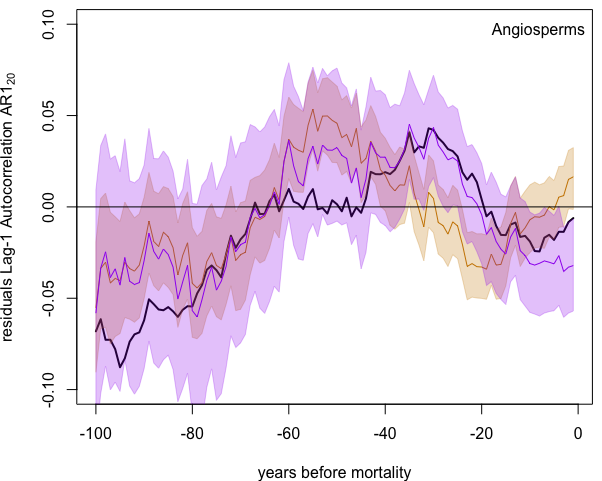
**
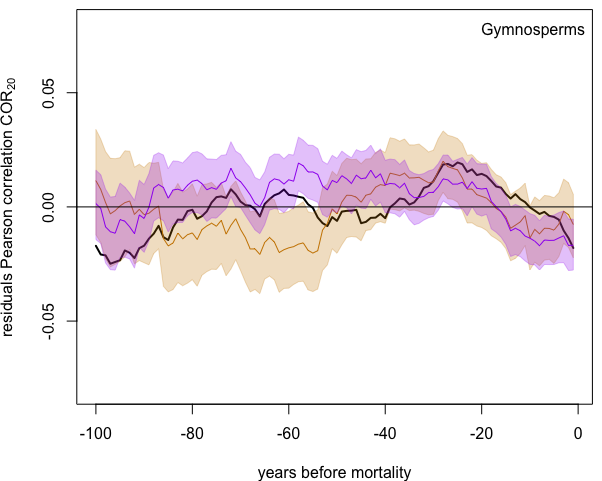

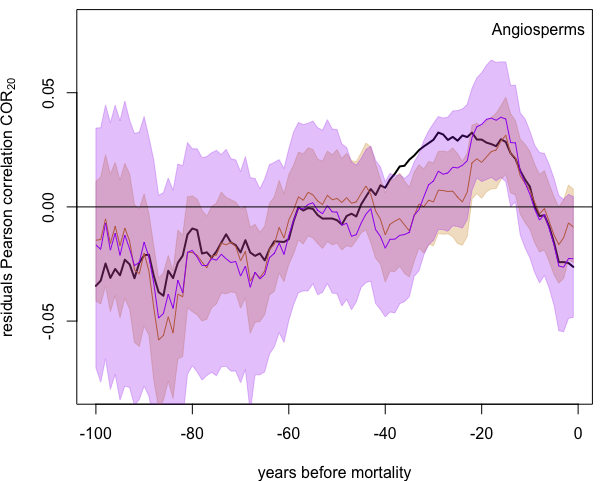


**(d)**

**(f)**

**(e)**

**(c)**

**Figure G1**: Average temporal change in the residuals in SD_20_ (a, b), AR1_20_ (c, d), and COR_20_ (e, f) of dying Angiosperms (a, c, e) and Gymnosperms (b, d, f) calculated by averaging data from all dying trees (original dataset; black), from a similar number of dead trees per species (n=36*53 ‘trees’ per dataset; orange) or per site (n=10*199 ‘trees’ per dataset; purple). Shaded areas represent the 95% confidence intervals of the means calculated based on 500 resamplings.


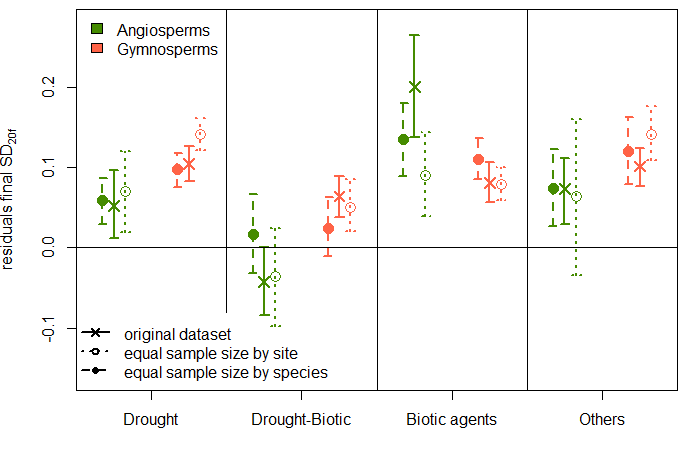


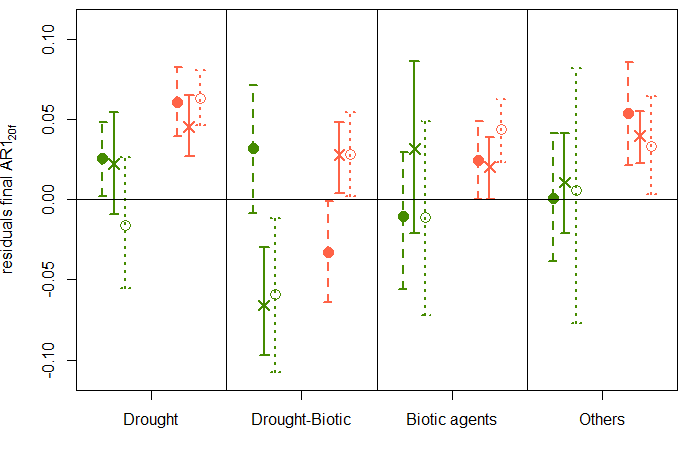

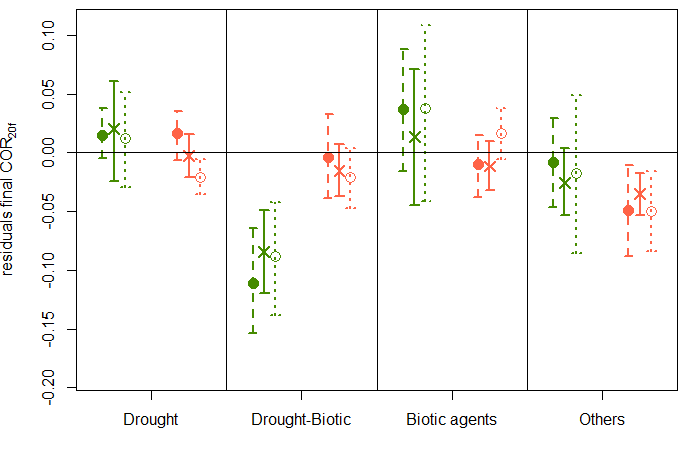


**Figure G2**: Variation in the residuals of SD (*a*), AR1 (*b*), and COR(*c*) calculated over the last 20-year period of the detrended RW time-series preceding tree death (resSD_20f_, resAR1_20f_, and resCOR_20f_) among mortality sources and species groups. Results for the original dataset are indicated with the crosses and full error bars. Medians and 95% confidence intervals of the mean residuals were determined based on the 500 datasets generated by resampling an equal number of dying trees per species (full dot and dashed error bar) or per site (open dot and dotted error bar).

**Similar mean RW**

**Similar DBH**

**
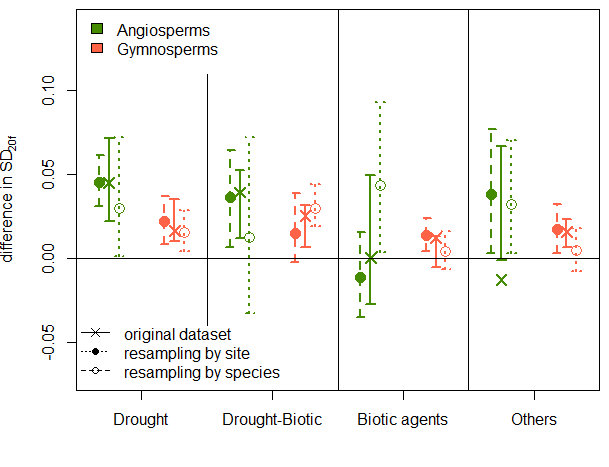

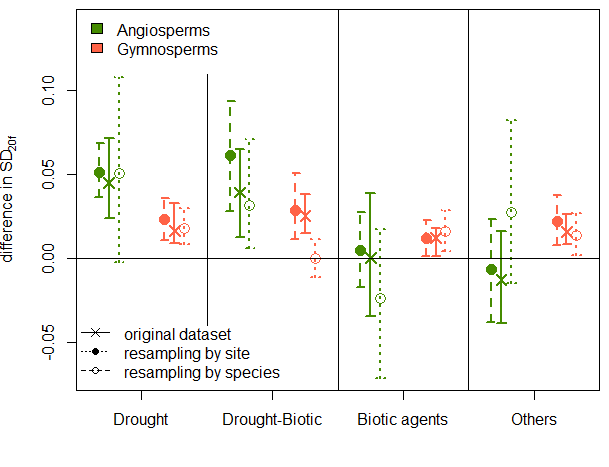
**

**(b)**

**(a)**

**
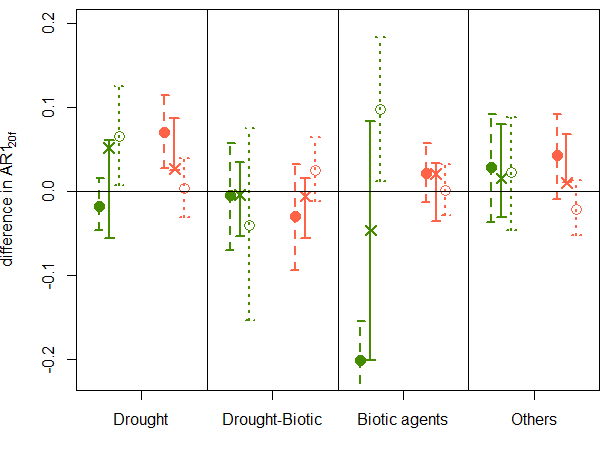

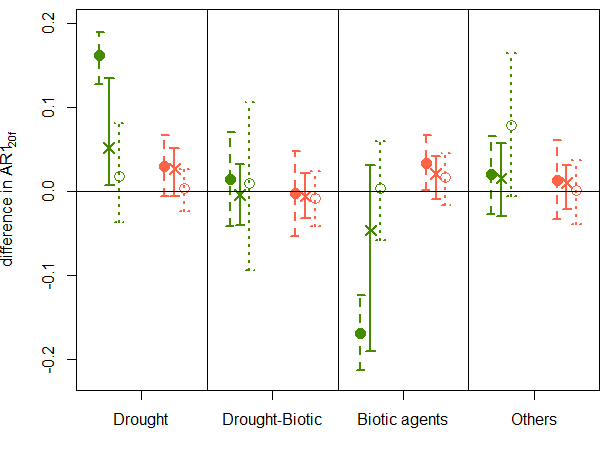

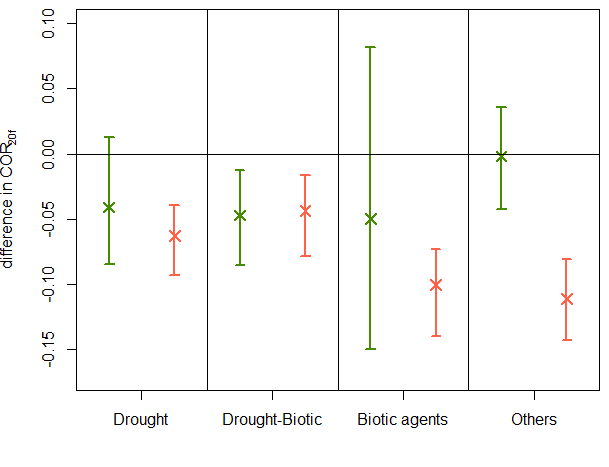

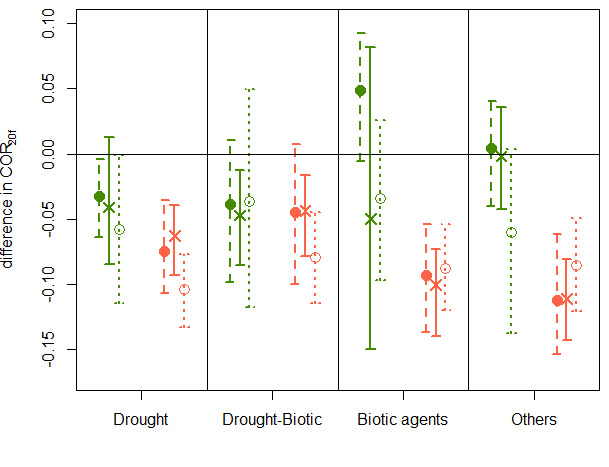
**

**(e)**

**(f)**

**(d)**

**(c)**

**Figure G3:** Mean difference in SD_20f_ (*Top*), AR1_20f_ (*Middle*), and COR_20f_ (*Bottom*) values between dying and surviving trees predicted by the LMMs fitted to the original dataset (crosses and full error bar), and to the 500 different datasets generated using a random sample of 34 or 47 dying/surviving pairs per species (full dot and dashed error bar) or a random sample of 7 or 8 pairs per site (white dot and dotted error bar) fixing diff_D-S_RW_20f_ and diff_D-S_DBH_f_ at zero. Standardization with similar meanRW_20f_ (*Left*) and similar DBH_f_ (*Right*). 95% of the predicted values from the 500 datasets are included within the confidence intervals (arrows). Estimates of the LMMs are available in Table H1. Note the extreme outliers observed for the differences in AR1 when the samples included an equal number of pairs for each species. The strong positive values calculated for angiosperms in the case of drought-induced mortality (pairing by DBH_f_; panel *d*) were attributed to *Quercus robur*, whose three pairs (with high diff_D-S_ AR1_20f_ values) proportionally have a higher weight than pairs of the other species. The same applies to angiosperms killed by biotic agents (due to very negative diff_D-S_ AR1_20f_ values of *Tamarix chinensis*).

**Appendix H: Summary of the linear mixed-effects models fitted to diff_D-S_ SD_20f_, diff_D-S_ AR1_20f_, and diff_D-S_ COR_20f_ values.**

**Table H1**: Summary of the linear mixed-effects models fitted to diff_D-S_ SD_20f_, diff_D-S_ AR1_20f_, and diff_D-S_ COR_20f_ values. In each cell we reported, the estimates and significance levels of regression coefficients of the LMMs fitted on the initial dataset (no-resampling; *Top*), and the estimates of the 95% confidence intervals of regression coefficients calculated based on LMM fitted to 500 different datasets generated using a random sample of 34 or 47 pairs per species (black square brackets; *Middle*), or a random sample of 7 or 8 pairs per site (grey square brackets; *Below*) with replacement (medians in the database for the standardization RW_20_ and DBH, respectively).

|  | diff_D-S_ SD_20f_ | | diff_D-S_ AR1_20f_ | | diff_D-S_ COR_20f_ | |
| --- | --- | --- | --- | --- | --- | --- |
|  | Similar RW_20_ | Similar DBH | Similar RW_20_ | Similar DBH | Similar RW_20_ | Similar DBH |
| Intercept | **0.047 ****  **[0.031 to 0.062]**  **[0.001 to 0.072]** | **0.045 ****  **[0.036 to 0.069]**  [-0.002 to 0.108] | 0.013  [-0.046 to 0.015]  **[0.007 to 0.125]** | 0.051  **[0.128 to 0.189]**  [-0.037 to 0.081] | -0.008  [-0.010 to 0.075]  **[-0.148 to -0.039]** | -0.041  **[-0.064 to -0.004]**  **[-0.114 to -0.001]** |
| diff_D-S_meanRW_20f_ | 0.20 (*)  [-0.209 to 0.617]  [-0.237 to 0.482] | **0.235 *****  **[0.235 to 0.278]**  **[0.196 to 0.229]** | 0.119  [-0.095 to 1.572]  [-0.221 to 1.104] | 0.018 (*)  **[0.012 to 0.070]**  [-0.014 to 0.028] | 0.168  [-0.994 to 0.226]  [-0.828 to 0.525] | **0.092 *****  **[0.069 to 0.132]**  **[0.041 to 0.087]** |
| diff_D-S_DBH_f_ | **-0.001 *****  **[-0.002 to -0.001]**  **[-0.001 to -0.001]** | -0.003  **[-0.013 to -0.001]**  [-0.001 to 0.018] | -0.000  [-0.002 to 0.001]  [-0.001 to 0.001] | **0.012 ***  **[0.008 to 0.037]**  **[0.012 to 0.039]** | **0.003 *****  **[0.001 to 0.003]**  **[0.002 to 0.004]** | **-0.012 ***  **[-0.034 to -0.002]**  **[-0.038 to -0.006]** |
| Drought-Biotic | -0.016  [-0.046 to 0.025] [-0.084 to 0.054] | -0.006  [-0.026 to 0.041]  [-0.072 to 0.035] | -0.024  [-0.059 to 0.082]  [-0.229 to 0.024] | -0.055  **[-0.214 to -0.084]**  [-0.125 to 0.092] | -0.026  [-0.136 to 0.001]  [-0.077 to 0.153] | -0.006  [-0.070 to 0.051]  [-0.272 to 0.111] |
| Biotic | -0.021  **[-0.084 to -0.028]** [-0.034 to 0.059] | -0.045  **[-0.078 to -0.019]**  [-0.172 to 0.009] | -0.020  **[-0.235 to -0.128]**  [-0.068 to 0.137] | -0.098  **[-0.383 to -0.273]**  [-0.090 to 0.057] | -0.114  [-0.044 to 0.072]  [-0.077 to 0.111] | -0.009  **[0.018 to 0.135]**  [-0.058 to 0.105] |
| Others | -0.019  [-0.048 to 0.033] [-0.037 to 0.041] | **-0.058 ****  **[-0.094 to -0.026]**  [-0.091 to 0.046] | 0.014  [-0.028 to 0.116]  [-0.133 to 0.039] | -0.036  **[-0.198 to -0.083]**  [-0.037 to 0.147] | -0.064  **[-0.183 to -0.052]**  [-0.045 to 0.097] | 0.039  [-0.014 to 0.135]  [-0.101 to 0.080] |
| Gymnosperms | -0.025  **[-0.044 to -0.003]** [-0.056 to 0.018] | -0.029 (*)  **[-0.047 to -0.011]**  [-0.087 to 0.021] | 0.041  **[0.035 to 0.145]**  [-0.130 to 0.009] | -0.025  **[-0.177 to -0.083]**  [-0.087 to 0.046] | -0.048  **[-0.151 to -0.046]**  [-0.027 to 0.097] | -0.022  [-0.087 to 0.004]  [-0.116 to 0.010] |
| Drought-Biotic * Gymno | 0.014  [-0.039 to 0.045] [-0.036 to 0.098] | 0.015  [-0.042 to 0.034]  [-0.056 to 0.055] | -0.052  **[-0.216 to -0.013]**  [-0.007 to 0.245] | 0.022  **[0.027 to 0.204]**  [-0.113 to 0.120] | 0.035  [-0.018 to 0.185]  [-0.215 to 0.048] | 0.025  [-0.062 to 0.125]  [-0.095 to 0.106] |
| Biotic * Gymno | 0.047  **[0.017 to 0.080]**  [-0.075 to 0.026] | 0.041  **[0.006 to 0.069]**  [-0.013 to 0.162] | -0.037  **[0.053 to 0.218]**  [-0.147 to 0.076] | 0.092  **[0.266 to 0.400]**  [-0.049 to 0.117] | 0.104  [-0.079 to 0.077]  [-0.087 to 0.109] | -0.029  **[-0.177 to -0.003]**  [-0.097 to 0.082] |
| Others * Gymno | 0.011  [-0.046 to 0.044] [-0.058 to 0.032] | **0.057 ***  **[0.025 to 0.097]**  [-0.053 to 0.088] | -0.042  [-0.178 to 0.027]  [-0.081 to 0.111] | 0.019  **[0.045 to 0.202]**  [-0.154 to 0.048] | 0.062  **[0.020 to 0.220]**  [-0.127 to 0.077] | -0.087  [-0.141 to 0.005]  [-0.067 to 0.126] |

The intercept corresponds to the reference species group (Angiosperms) and the reference mortality source (drought). In consequence, model estimates should be interpreted carefully. For instance, because of the high diff_D-S_ AR1_20f_ values for the condition ‘Angiosperms – drought-induced mortality – equivalent number of pairs for each species’, the estimates of the other categorical variables significantly differ from zero, which should not be interpreted as significant differences between diff_D-S_ AR1_20f_ and zero, but rather as significant differences between diff_D-S_ AR1_20f_ calculated for the given variable and for ‘drought-angiosperms’.

1. Cook, E. R. (1987). The decomposition of tree-ring series for environmental studies. *Tree-Ring Bulletin*. 47, 37-59 [↑](#footnote-ref-1)
